# Consistent supra­molecular motif of *C*(7) O—H⋯O hydrogen-bonded chains and different local symmetries in three iso­indole-4-carb­oxy­lic acid derivatives

**DOI:** 10.1107/S2056989025010709

**Published:** 2026-01-01

**Authors:** Kseniia A. Alekseeva, Atash V. Gurbanov, Ekaterina N. Tsiulina, Alexandra S. Golubenkova, Mehmet Akkurt, Gizachew Mulugeta Manahelohe

**Affiliations:** a RUDN University, 6 Miklukho-Maklaya St., Moscow 117198, Russian Federation; b Excellence Center, Baku State University, Z. Khalilov Str., AZ 33, Baku, Azerbaijan; c Department of Physics, Faculty of Sciences, Erciyes University, 38039 Kayseri, Türkiye; d Department of Chemistry, University of Gondar, PO Box 196, Gondar, Ethiopia; University of Aberdeen, United Kingdom

**Keywords:** crystal structure, hydrogen bonds, zigzag chains, Hirshfeld surface analysis

## Abstract

The syntheses and structures of (3a*RS*,4*RS*,9a*SR*)-3-oxo-2-(2-phenyl­eth­yl)-2,3,3a,4,9,9a-hexa­hydro-1*H*-benzo[*f*]iso­indole-4-carb­oxy­lic acid, C_21_H_21_NO_3_ (I), (3a*RS*,4*RS*,9a*SR*)-3-oxo-2-(propan-2-yl)-2,3,3a,4,9,9a-hexa­hydro-1*H*-benzo[*f*]iso­indole-4-carb­oxy­lic acid, C_16_H_19_NO_3_ (II) and (4*RS*)-3-oxo-2-phenyl-2,3,4,9-tetra­hydro-1*H*-benzo[*f*]iso­indole-4-carb­oxy­lic acid, C_19_H_15_NO_3_ (III), are described. Compound (I) crystallizes with two mol­ecules in the asymmetric unit. In the extended structures of (I), (II) and (III) the mol­ecules are linked by O_c_—H⋯O_i_ (c = carb­oxy­lic acid, i = indole) hydrogen bonds, forming a common *C*(7) zigzag chain motif propagating along the [010] direction, despite the different local symmetries. These chains are connected by weak C—H⋯O and C—H⋯π inter­actions, forming a three-dimensional network in each case. A Hirshfeld surface analysis was undertaken to investigate and qu­antify the inter­molecular inter­actions, which are dominated by H⋯H, C⋯H/H⋯C and O⋯H/H⋯O contacts in each case.

## Abstract 2

The title compounds exhibit a consistent pattern of inter­molecular O—H⋯O hydrogen bonds, forming a *C*(7) zigzag chain propagating in the [010] direction in each case.

## 1. Chemical context

The iso­indole motif is a common structural element found in numerous natural and biologically active compounds. This fragment occurs in cyclo­piazonic acid, cytochalasan and anthra­quinone alkaloids, as well as in meroterpenoids of the *Stachybotrys* species (Speck & Magauer, 2013 ▸). Compounds containing the iso­indole skeleton exhibit a wide range of biological activities, including anti­cancer and anti­microbial properties (Bhatia, 2016 ▸).

Among the various synthetic approaches used to construct the iso­indole core, the intra­molecular Diels–Alder reaction of vinyl­arenes (IMDAV) has emerged as a particularly powerful method (see the recent review by Krishna *et al.*, 2022 ▸). This reaction, as an extension of the traditional Diels–Alder methodology, provides an efficient route to complex polycyclic aromatic and carbocyclic systems. In the IMDAV reaction, the vinyl­arene moiety acts as the dienophile, enabling the formation of fused or bridged ring structures with remarkable regio- and stereocontrol. By positioning the reactive partners within the same mol­ecule, the IMDAV process overcomes the typically low reactivity of vinyl­arenes observed in inter­molecular Diels–Alder reactions, thereby enhancing both reaction rates and selectivity. This transformation is especially valuable for constructing the iso­indole scaffold, including partially hydrogenated iso­indoles fused with benzene (Voronov *et al.*, 2018 ▸), furan (Fischer & Hünig, 1987 ▸; Alekseeva *et al.*, 2020 ▸), or thio­phene (Herz *et al.*, 2001 ▸; Nadirova *et al.*, 2020 ▸) rings.

The significance of the IMDAV reaction lies in its broad utility for the synthesis of natural products, pharmaceuticals, and advanced materials. Many biologically active compounds contain fused aromatic frameworks or rigid polycyclic skeletons that can be efficiently accessed through IMDAV strategies. Furthermore, the ability to incorporate aromatic functionality directly into the newly formed ring systems provides unique opportunities for subsequent functionalization and mol­ecular diversification. It is noteworthy that the target iso­indoles described in this work are structurally related to compounds exhibiting selective inhibitory activity toward PTP (De Cesco *et al.*, 2012 ▸) and PARP enzymes (Papeo *et al.*, 2015 ▸).

In this study, we report the syntheses and structures of three new iso­indole derivatives (3a*RS*,4*RS*,9a*SR*)-3-oxo-2-(2-phenyl­eth­yl)-2,3,3a,4,9,9a-hexa­hydro-1*H*-benzo[*f*]iso­indole-4-carb­oxy­lic acid, C_21_H_21_NO_3_ (I), (3a*RS*,4*RS*, 9a*SR*)-3-oxo-2-(propan-2-yl)-2,3,3a,4,9,9a-hexa­hydro-1*H*-benzo[*f*]iso­indole-4-carb­oxy­lic acid, C_16_H_19_NO_3_ (II) and (4*RS*)-3-oxo-2-phenyl-2,3,4,9-tetra­hydro-1*H*-benzo[*f*]iso­indole-4-carb­oxy­lic acid, C_19_H_15_NO_3_ (III). Compounds (I)–(III) are obtainable from readily available precursors – secondary amines and maleic anhydrides. The proposed synthetic approach does not require strictly anhydrous solvents or inert atmospheres and enables a practically one-pot, diastereoselective formation of complex polycyclic systems.
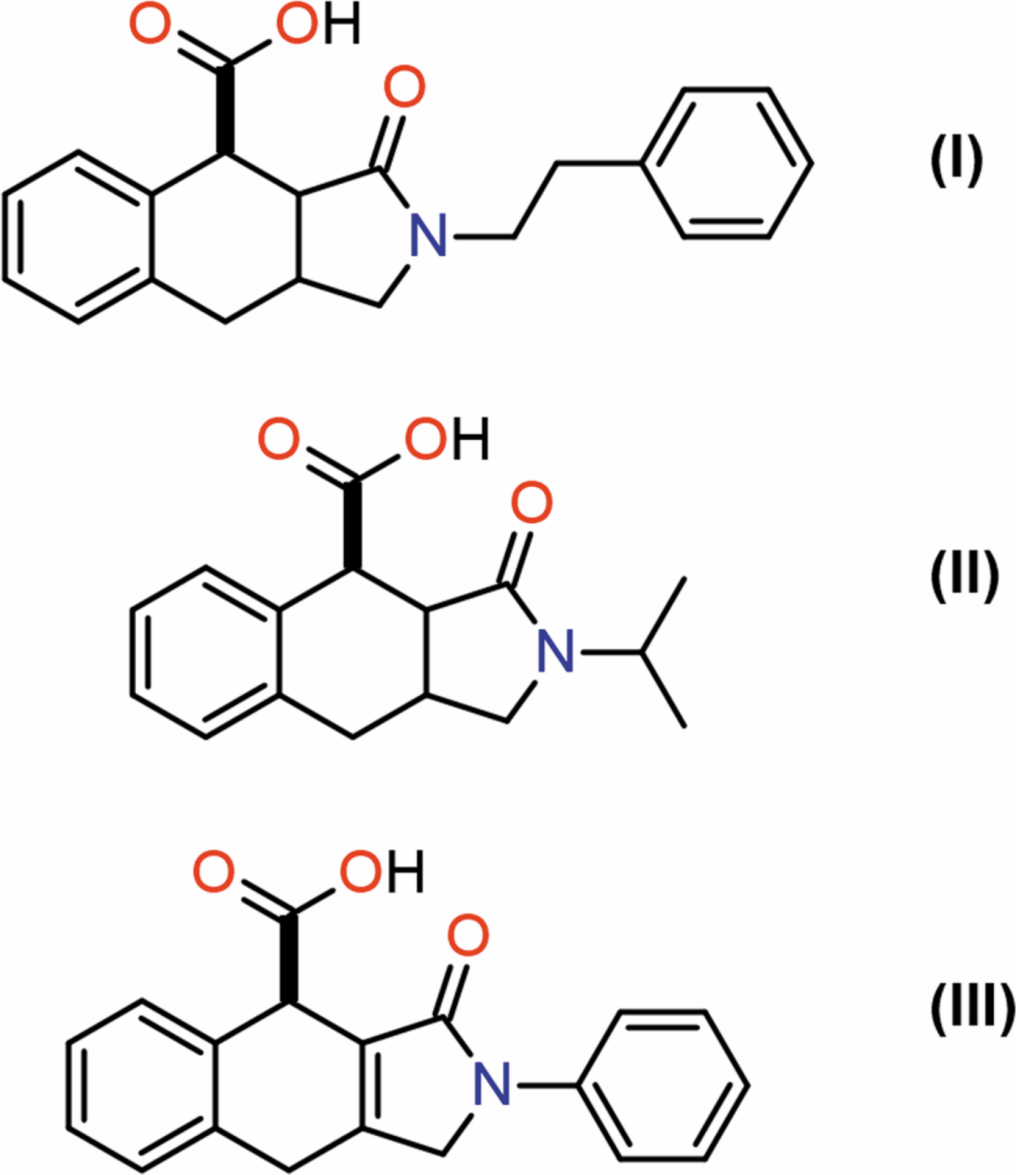


## 2. Structural commentary

**Figure 1:**
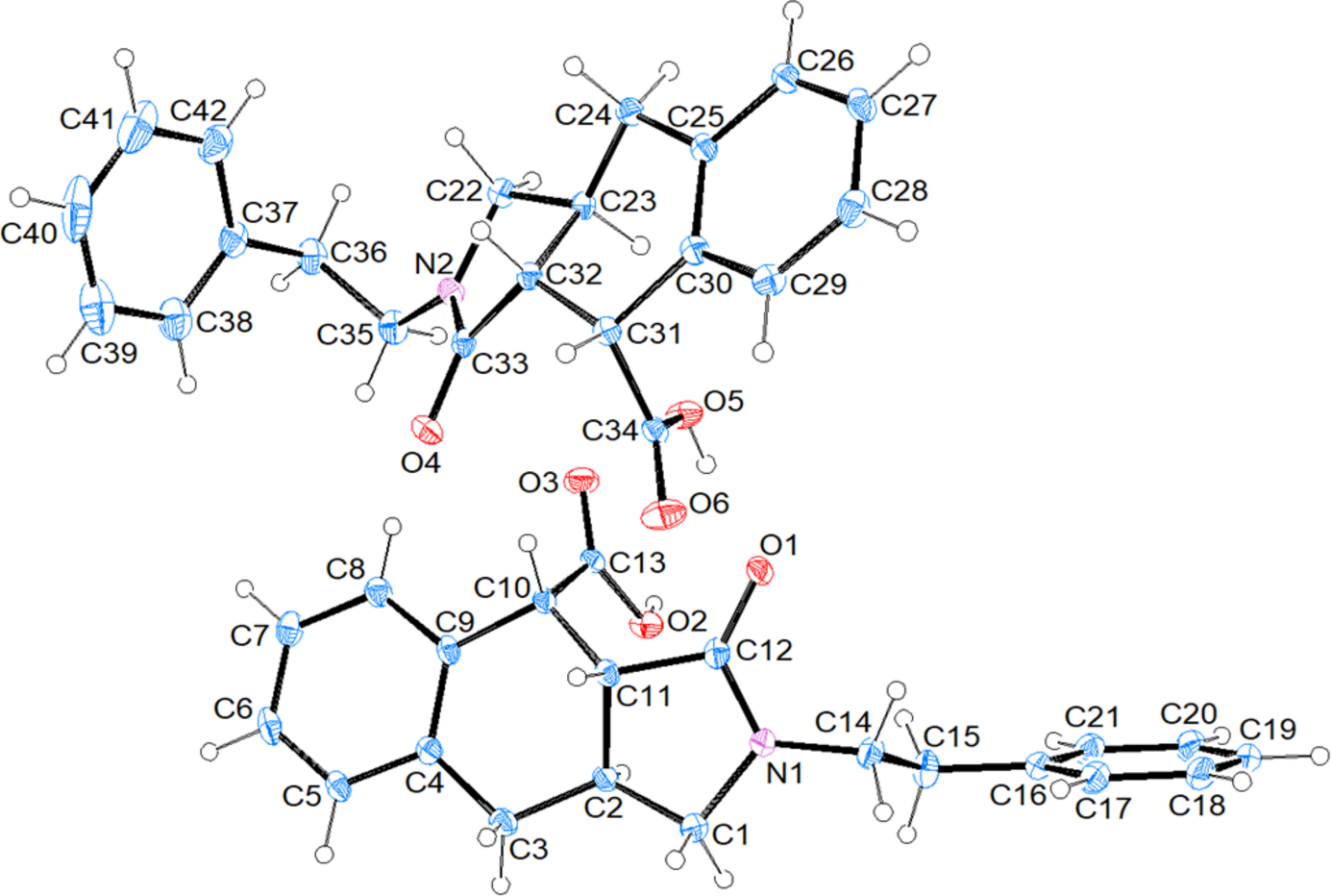
The mol­ecular structure of (I) with displacement ellipsoids drawn at the 50% probability level.

**Figure 2:**
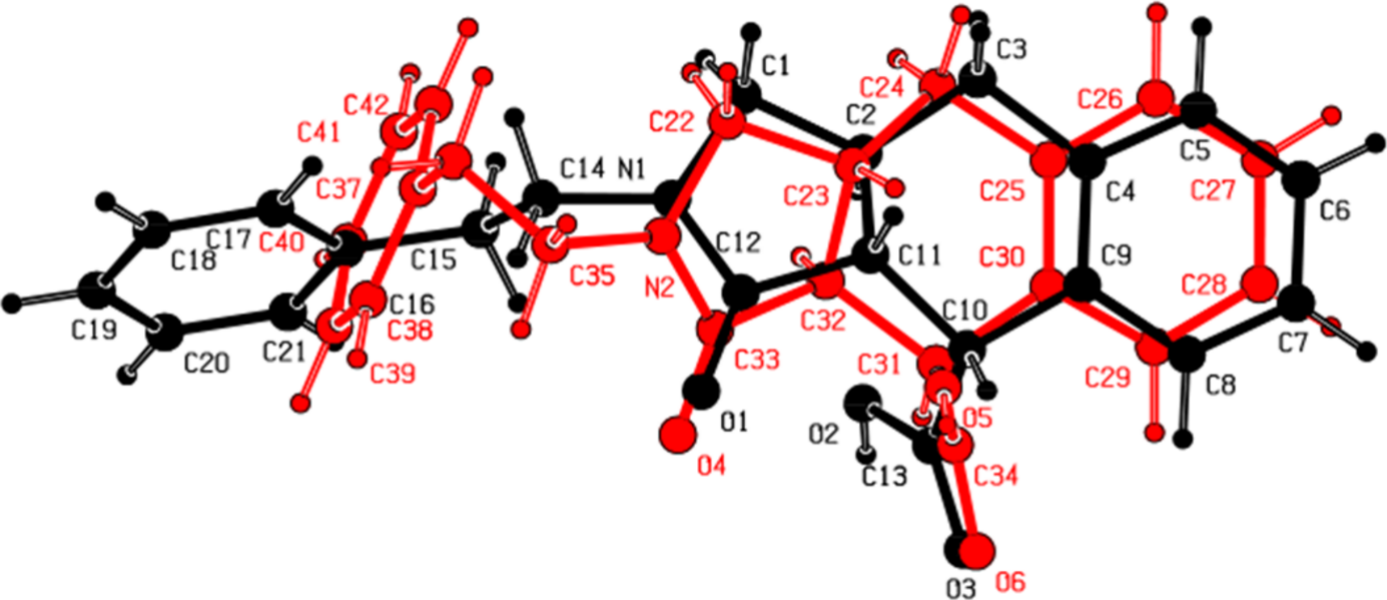
A least-squares overlay of the two independent mol­ecules (*A* black and *B* red) in (I).

The asymmetric unit of compound (I) (space group *P*2_1_/*n*) consists of two mol­ecules (*A* containing N1 and *B* containing N2) (Fig. 1 ▸). Each mol­ecule contains three stereogenic (chiral) centres: in the arbitrarily chosen asymmetric unit, C2, C10 and C11 have *S*, *R* and *R* configurations, respectively, in mol­ecule *A* and C23, C31 and C32 have *S*, *R* and *R* configurations, respectively, in mol­ecule *B*. In mol­ecule *A* (Fig. 1 ▸), the least-squares plane (r.m.s. deviation = 0.002 Å) of the thirteen-membered ring system (N1/C1–C12) indicates near planarity and it makes a dihedral angle of 75.11 (10)° with the terminal phenyl ring (C16–C21). The 2,5-di­hydro-1*H*-pyrrole ring (N1/C1/C2/C11/C12) adopts an envelope conformation on atom C2 with puckering parameters (Cremer & Pople, 1975 ▸) *Q*(2) = 0.344 (3) Å, φ(2) = 258.4 (4)° and the cyclo­hexa-1,4-diene ring (C2–C4/C9–C11) exhibits a twist-boat conformation [the puckering parameters are *Q*_T_ = 0.508 (3) Å, θ = 127.6 (3) °, φ = 161.9 (4)°]. In mol­ecule *B* (Fig. 1 ▸), the best plane (r.m.s. deviation = 0.002 Å) of the thirteen-membered ring system (N2/C22–C33) makes a dihedral angle of 80.86 (10)° with the terminal phenyl ring (C37–C42). The 2,5-di­hydro-1*H*-pyrrole ring (N2/C22/C23/C32/C33) adopts an envelope conformation on atom C23 with puckering parameters of *Q*(2) = 0.356 (2) Å, φ(2) = 253.2 (4)°, and the cyclo­hexa-1,4-diene ring (C23–C25/C30–C32) exhibits a twist-boat conformation with puckering parameters *Q*_T_ = 0.523 (2) Å, θ = 128.8 (2)° and φ = 164.9 (3)°. The major difference between *A* and *B* is seen in the N_i_—C—C—C_ar_ (i = indole, ar = aromatic) torsion angles, being 171.36 (19)° (*anti*) for mol­ecule *A* and −61.4 (3)° (*gauche*) for *B*. An overlay fit of the inverted mol­ecule *B* on mol­ecule *A* is shown in Fig. 2 ▸ with the weighted r.m.s. fit of the 25 non-H atoms being 2.34 Å.

**Figure 3:**
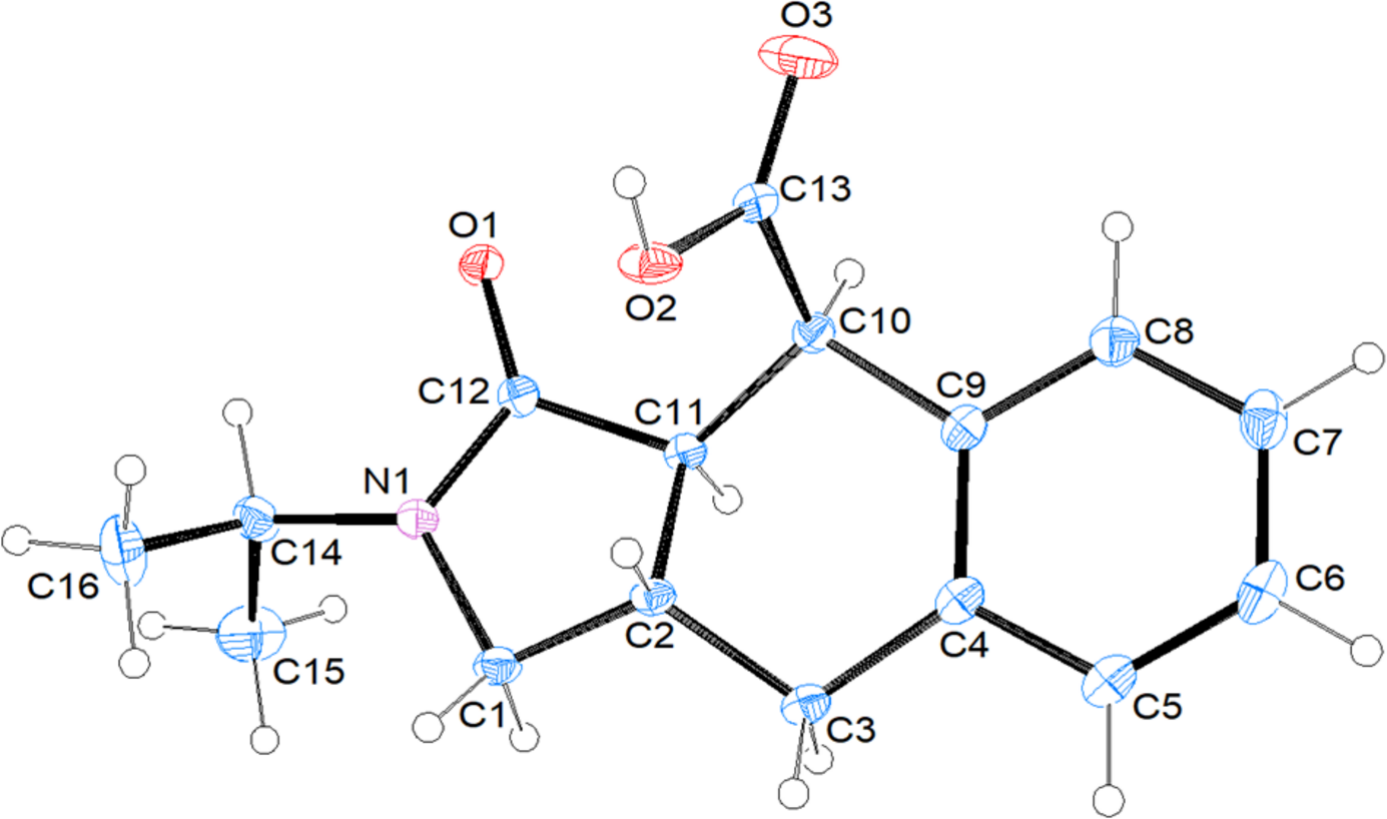
The mol­ecular structure of (II), showing displacement ellipsoids drawn at the 50% probability level.

Compound (II) crystallizes in space group *C*2/*c* with one mol­ecule in the asymmetric unit (Fig. 3 ▸). The stereogenic centres in the arbitrarily chosen asymmetric mol­ecule – C2, C10 and C11 – have *S*, *R* and *R* configurations, respectively, but crystal symmetry generates a racemic mixture. The thirteen-membered ring system (N1/C1–C12) is essentially planar (r.m.s. deviation = 0.002 Å). The 2,5-di­hydro-1*H*-pyrrole ring (N1/C1/C2/C11/C12) adopts an envelope conformation on atom C2 with puckering parameters of *Q*(2) = 0.3634 (17) Å and φ(2) = 255.1 (3)°, and the cyclo­hexa-1,4-diene ring (C2–C4/C9–C11) has a twist-boat conformation [the puckering parameters are *Q*_T_ = 0.4941 (17) Å, θ = 132.0 (2)°, φ = 157.7 (3)°].

**Figure 4:**
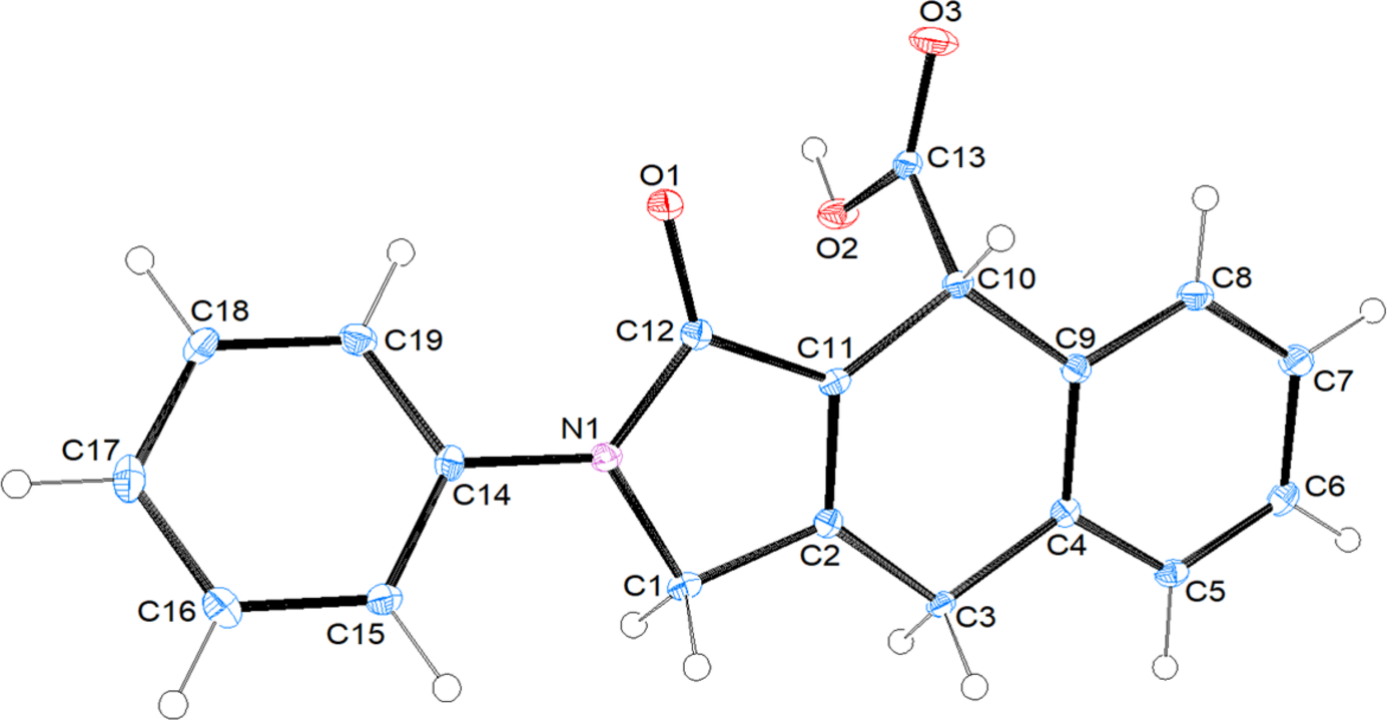
The mol­ecular structure of (III), showing displacement ellipsoids drawn at the 50% probability level.

In (III), which crystallizes in space group *P*2_1_/*c* (Fig. 4 ▸), the thirteen-membered ring system (N1/C1–C12) is essentially planar (r.m.s. deviation = 0.002 Å) and makes a dihedral angle of 32.15 (9)° with the terminal phenyl ring (C14–C19). The sole stereogenic centre, C10, has an *R* configuration in the arbitrarily chosen asymmetric unit, but crystal symmetry generates a racemic mixture. The 2,5-di­hydro-1*H*-pyrrole ring (N1/C1/C2/C11/C12) and the cyclo­hexa-1,4-diene ring (C2–C4/C9–C11) are essentially planar with maximum deviations of −0.023 (2) Å for N1 and 0.012 (2) Å for C9, respectively. Otherwise, the bond lengths and angles in the mol­ecules of (I), (II) and (III) are comparable with each other and with those of related structures detailed in the *Database survey* (section 4).

## 3. Supra­molecular features and Hirshfeld surface analysis

**Figure 5:**
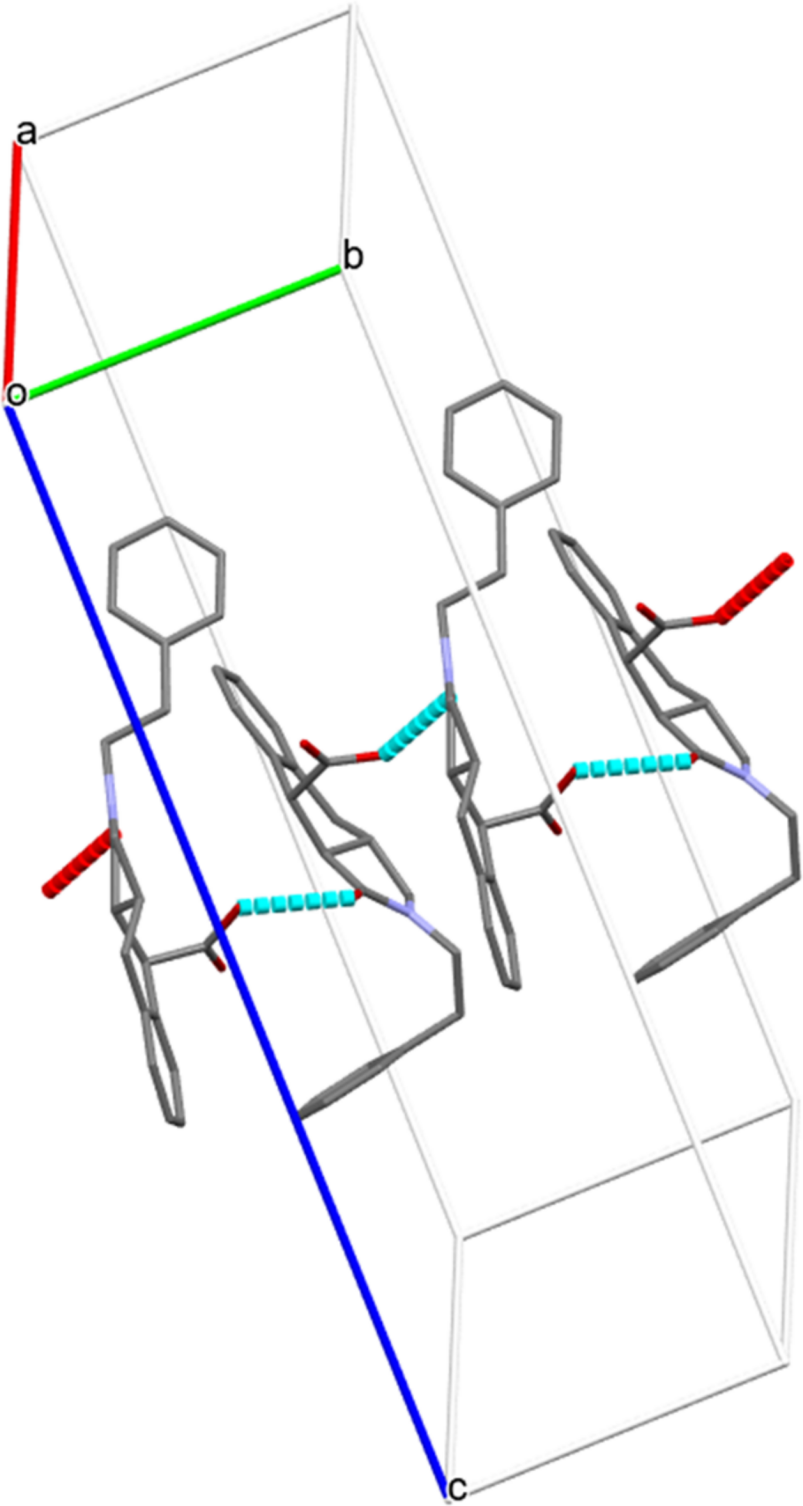
Partial packing diagram for (I) showing O—H⋯O hydrogen bonds forming *C*(7) zigzag chains propagating along the [010] direction. The remaining hydrogen atoms are omitted for clarity.

In the extended structures of (I), (II), and (III), the mol­ecules are linked by a consistent O—H⋯O hydrogen bond from the carb­oxy­lic acid OH group to the indole O atom, forming a *C*(7) zigzag chain propagating along the [010] direction in each case (Tables 1 ▸, 2 ▸ and 3 ▸; Figs. 5 ▸, 6 ▸ and 7 ▸). Thus, it may be noted that carb­oxy­lic acid homodimers linked by pairs of O—H⋯O hydrogen bonds are *not* formed. In (I), the *A* and *B* mol­ecules alternate in the chain and alternate mol­ecules of each kind are related by simple unit-cell translation in the *b*-axis direction. In (II) and (III), adjacent mol­ecules in the chain are related by a 2_1_ screw axis. These chains are connected by various C—H⋯O and C—H⋯π inter­actions, forming three-dimensional networks in each case (Figs. S1–S9 in the supporting information).

**Figure 8:**
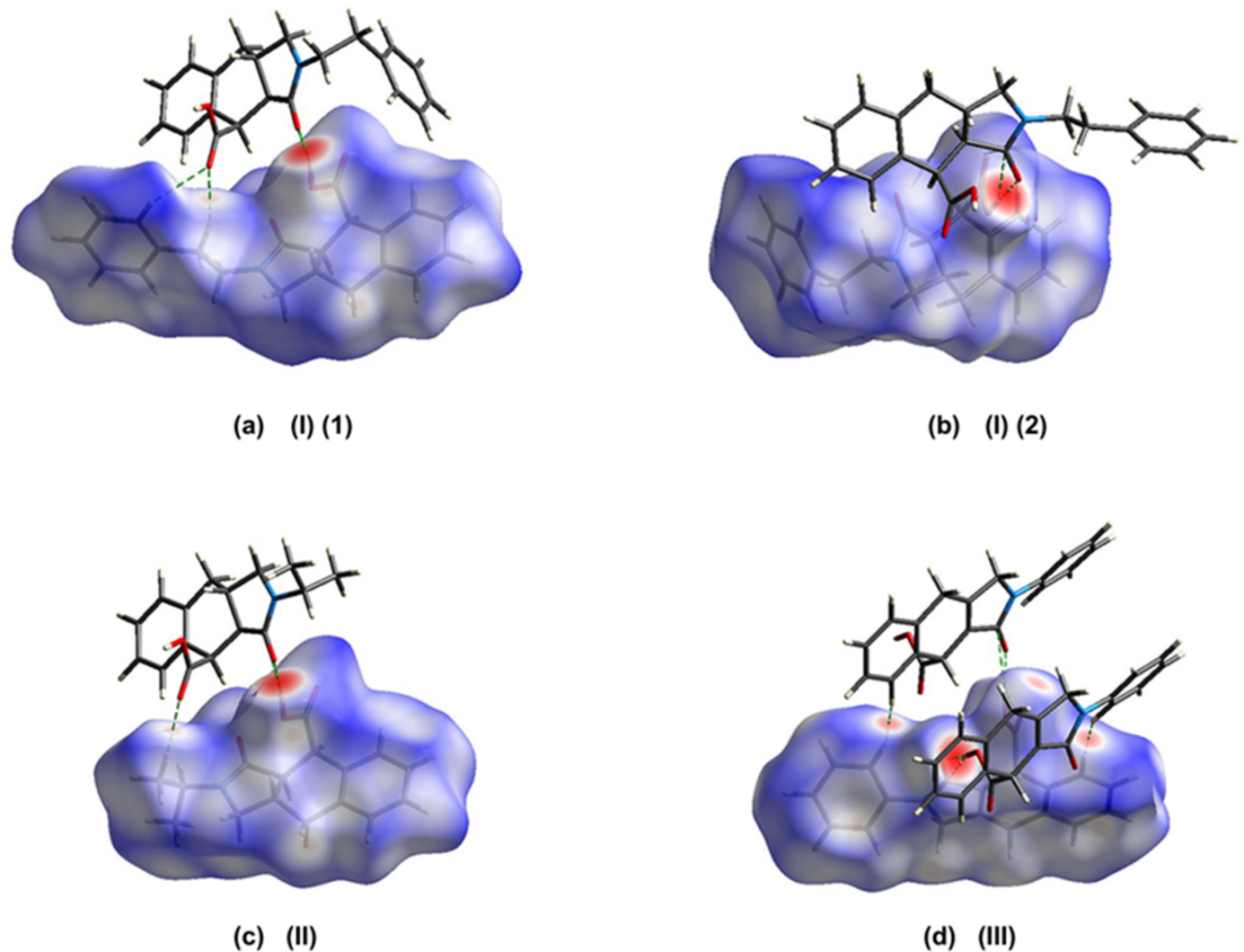
Views of the three-dimensional Hirshfeld surfaces of the compounds (I)*A*, (I)*B*, (II) and (III) mapped over *d*_norm_.

**Figure 9:**
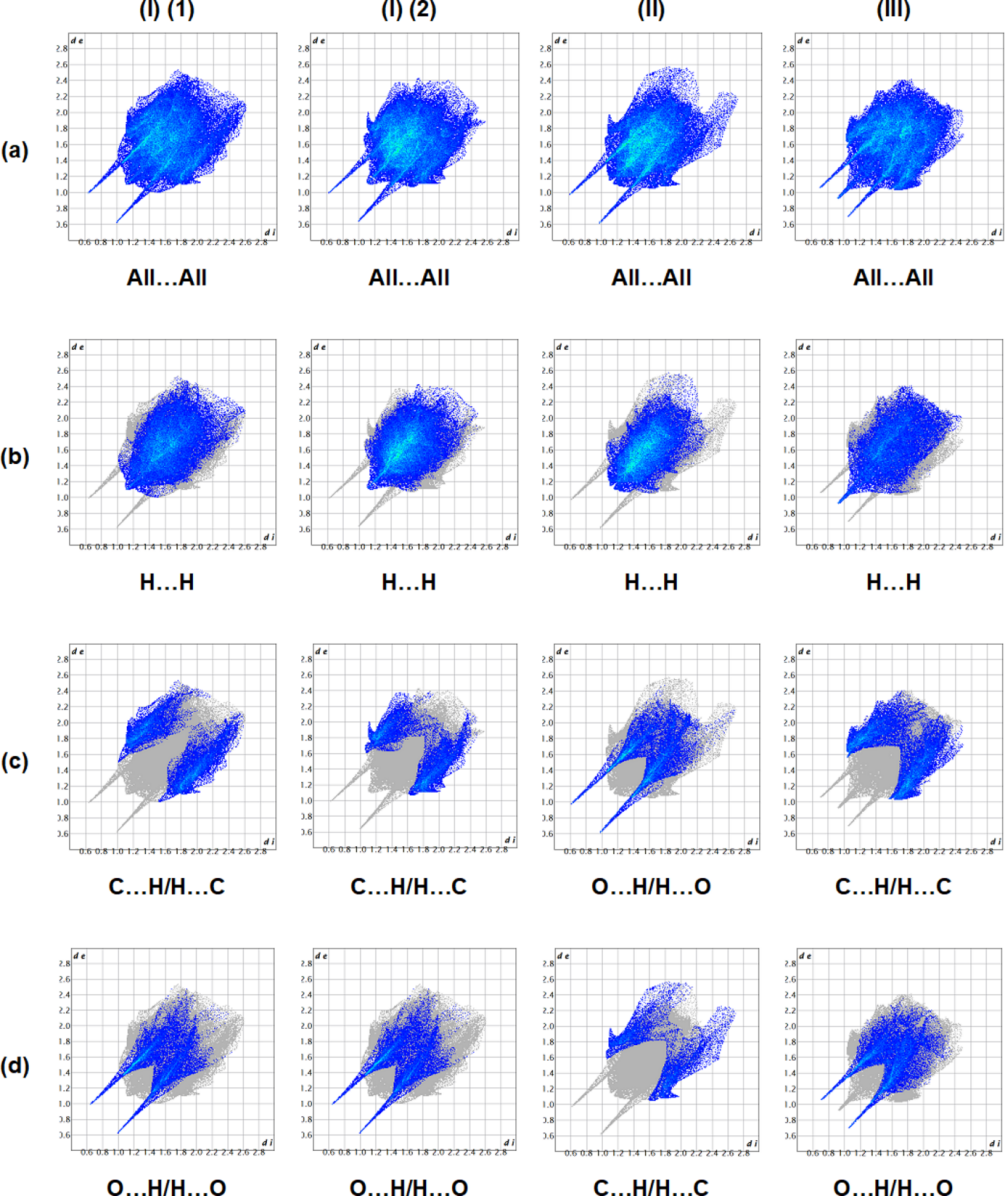
A view of the two-dimensional fingerprint plots for compounds (I)*A*, (I)*B*, (II) and (III), showing (*a*) all inter­actions, and delineated into (*b*) H⋯H, (*c*) C⋯H/H⋯C for (I)*A*, (I)*B* and (III), and O⋯H/H⋯O for (II) and (*d*) O⋯H/H⋯O for (I)*A*, (I)*B* and (III), and C⋯H/H⋯C for (II) inter­actions.

The Hirshfeld surfaces and corresponding two-dimensional fingerprint plots were calculated using *Crystal Explorer 17.5* (Spackman *et al.*, 2021 ▸) to further qu­antify the inter­molecular inter­actions. The *d*_norm_ mappings for mol­ecules (I)*A*, (I)*B*, (II) and (III) were performed in the ranges −0.76 to +1.46 a.u., −0.76 to +1.46 a.u., −0.78 to +1.41 a.u. and −0.66 to +1.21 a.u., respectively. The O—H⋯O and C—H⋯O inter­actions are indicated by red areas on the Hirshfeld surfaces; see Fig. 8 ▸*a* for (I)*A*, Fig. 8 ▸*b* for (I)*B*, Fig. 8 ▸*c* for (II), and Fig. 8 ▸*d* for (III). The two-dimensional fingerprint plots are given in Fig. 9 ▸, where H⋯H, C⋯H/H⋯C and O⋯H/H⋯O inter­actions dominate for all three structures with the other contact types making a negligible contribution (Table 4 ▸).

## 4. Database survey

A Cambridge Structural Database (CSD, Version 6.00, last update April 2025; Groom *et al.*, 2016 ▸) search indicated that the five most similar compounds to the title compounds containing the 2,3,4,5,6,7-hexa­hydro-1*H*-iso­indole unit are CSD refcodes EHURIM (Yakovleva *et al.*, 2025 ▸), OJIPUV (Zaytsev *et al.*, 2021 ▸), TODKEF (Elliott & Booker-Milburn, 2019 ▸), BAFYAL (Zhong *et al.*, 2017 ▸) and QAFSUO (Zubkov *et al.*, 2016 ▸). In the crystal of EHURIM, the mol­ecules are connected by C—H⋯O hydrogen bonds, forming layers lying parallel to the (101) plane. Furthermore, the mol­ecules form layers parallel to the (10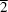) plane by way of C—H⋯π inter­actions. In In OJIPUV the mol­ecules are connected by C—H⋯O hydrogen bonds, C—H⋯π inter­actions and π–π stacking inter­actions, forming a three-dimensional network. In TODKEF, the mol­ecules are linked by C—H⋯O and O—H⋯O hydrogen bonds, forming a three-dimensional network with C—H⋯π inter­actions also observed. In BAFYAL, the mol­ecules are linked by C—H⋯O inter­actions, forming layers lying parallel to the (002) plane; π–π inter­actions are also present. In QAFSUO, the three-dimensional packing is consolidated by O—H⋯O and C—H⋯O contacts and C—H⋯π inter­actions.

## 5. Synthesis and crystallization

To prepare (I), (2*E*)-3-phenyl-*N*-(2-phenyl­eth­yl)prop-2-en-1-amine (0.73 g, 3.06 mmol) was dissolved in 1,4-dioxane (15 ml) and maleic anhydride (0.30 g, 3.06 mmol) was added. The reaction mixture was stirred for 30 min; after that 4-(di­methyl­amino)­pyridine (0.75 g, 6.12 mmol) was added. The resulting solution was refluxed for 8 h. After cooling to room temperature, the reaction mixture was poured into water (30 ml) and acidified with concentrated hydro­chloric acid to pH ∼6. The aqueous phase was extracted with ethyl acetate (3 × 10 ml). The combined organic layers were dried over sodium sulfate, filtered, and concentrated under reduced pressure. Upon addition of ethyl acetate to the residue, a white precipitate formed, which was collected by filtration and air-dried to afford the target product as a white solid (0.53 g, 1.60 mmol, 52%, m.p. 510–513 K). Single crystals of (I) suitable for X-ray diffraction analysis were grown from the mixed solvents of ethanol and di­methyl­formamide.

^1^H NMR (600 MHz, DMSO-*d*_6_, 293 K) (*J*, Hz): *δ* 12.45 (*br. s*, 1H, COOH), 7.44–7.43 (*m*, 1H, H-Ar), 7.30–7.28 (*m*, 2H, H-Ar), 7.26–7.25 (*m*, 2H, H-Ar), 7.22–7.16 (*m*, 4H, H-Ar), 3.94 (*d*, *J =* 6.1, 1H, H-Ar), 3.49–3.45 (*m*, 1H, H-NCH_2_CH_2_), 3.42–3.37 (*m*, 2H, H-NCH_2_CH_2_, H-1A), 3.08 (*dd*, *J =* 9.1, 9.6, 1H, H-1), 2.99–2.91 (*m*, 2H, H-3a,9a), 2.80–2.73 (*m*, 2H, H-NCH_2_CH_2_), 2.66 (*br. dd*, *J =* 12.6, 16.1, 1H, H-9A), 2.38 (*dd*, *J =* 5.5, 12.6, 1H, H-9B) ppm. ^13^C {^1^H} NMR (151 MHz, DMSO-*d*_6_, 296 K) *δ* 173.0, 172.8, 139.2, 136.6, 133.0, 130.0, 129.9, 128.7 (2C), 128.4 (2C), 127.0, 126.2, 126.1, 51.0, 46.1, 43.6, 42.4, 33.4, 32.8, 32.0 ppm. IR (KBr), ν (cm^−1^) 3011 (COOH), 1715 (C=O), 1640 (NCO). Analysis calculated for C_21_H_21_NO_3_: C, 75.20; H, 6.31; N, 4.18. Found: C, 75.07; H, 6.44; N, 4.39.

To prepare (II), (2*E*)-3-phenyl-*N*-(propan-2-yl)prop-2-en-1-amine (0.53 g, 3.06 mmol) was dissolved in aceto­nitrile (15 ml), and maleic anhydride (0.30 g, 3.06 mmol) was added. The reaction mixture was refluxed for 8 h. Upon slowly cooling to room temperature, the product crystallized from the reaction mixture. The resulting solid was collected by filtration, washed with diethyl ether, and air-dried to afford the target compound as a white crystalline solid (0.57 g, 2.08 mmol, 68%, m.p. 502–503 K). A single crystal of (II) suitable for X-ray diffraction analysis was isolated from the obtained crystalline material.

^1^H NMR (600 MHz, DMSO-*d*_6_, 295 K) (*J*, Hz): *δ* 12.37 (*br. s*, 1H, COOH), 7.43 (*dd*, *J =* 2.1, 7.5, 1H, H-5-Ph), 7.21–7.17 (*m*, 3H, H-6,7,8-Ph), 4.17 (*hept*, *J =* 6.8, 1H, H-2-*i*-Pr), 3.93 (*dd*, *J =* 6.1, 1H, H-4), 3.55 (*dd*, *J =* 6.6, 8.6, 1H, H-1*A*), 3.00–2.89 (*m*, 3H, H-1*B*,9*A*,9a), 2.71 (*dd*, *J =* 12.1, 15.6, 1H, H-9*B*), 2.40 (*dd*, *J =* 6.1, 13.1, 1H, H-3a), 1.10 (*d*, *J =* 6.8, 3H, H-1-*i*-Pr), 1.07 (*d*, *J =* 6.8, 3H, H-3-*i*-Pr) ppm. ^13^C {^1^H} NMR (151 MHz, DMSO-*d*_6_, 296 K) *δ* 173.0, 172.1, 136.6, 133.2, 130.0, 127.0, 126.1, 46.5, 45.2, 42.4, 41.6, 32.8, 32.0, 19.9, 19.5 ppm. IR (KBr), ν (cm^−1^) 3000(OH), 1725 (C=O), 1644 (NCO). Analysis calculate. for C_16_H_19_NO_3_: C, 70.31; H, 7.01; N, 5.12. Found: C, 70.35; H, 6.93; N, 5.08.

Synthesis of (III): *N*-[(2*E*)-3-phenyl­prop-2-en-1-yl]aniline (0.28 g, 1.28 mmol) was dissolved in 1,4-dioxane (15 ml), and bromo­maleic anhydride (0.25 g, 1.41 mmol) was added. The reaction mixture was refluxed for 8 h. After cooling to room temperature, the solvent was removed under reduced pressure. Ethanol (3 ml) was then added to the residue, resulting in the formation of a precipitate of product, which was collected by filtration, washed with ethanol (2 × 1 ml), and air-dried to afford the target compound as a white solid (0.12 g, 0.38 mmol, 30%, m.p. 513–514 K). A single crystal of (III) suitable for X-ray diffraction analysis was grown from an EtOH/DMF solution.

^1^H NMR (600 MHz, DMSO-*d*_6_, 294 K) (*J*, Hz): *δ* 12.72 (*s*, 1H, COOH), 7.80 (*dd*, *J =* 1.0, 8.6, 2H, H-2,6-Ph), 7.53 (*dd*, *J =* 1.5, 7.1, 1H, H-5), 7.41–7.28 (*m*, 5H, H-6,7,8, H-3,5-Ph), 7.11 (*dt*, *J =* 1.0, 7.1, 1H, H-4-Ph), 4.75 (*d*, *J =* 18.7, 1H, H-1*A*), 4.61 (*d*, *J =* 2.0, 18.7, 1H, H-1*B*), 4.55–4.53 (*m*, 1H, H-4), 3.75 (*dd*, *J =* 4.0, 22.5, 1H, H-9*A*), 3.74 (*dd*, *J =* 4.0, 22.5, 1H, H-9*B*) ppm. ^13^C {^1^H} NMR (151 MHz, DMSO-*d*_6_, 296 K) *δ* 172.1, 168.3, 150.6, 139.5, 132.2, 131.1, 129.3, 129.0 (2C), 128.5, 128.4, 127.5, 126.8, 123.3, 118.0 (2C), 52.7, 42.6, 28.8 ppm. IR (KBr), ν (cm^−1^) 3042 (COOH), 1739 (C=O). Analysis calculated for C_19_H_15_NO_3_: C, 74.74; H, 4.95; N, 4.59. Found: C, 74.68; H, 5.03; N, 4.65.

## 6. Refinement

Crystal data, data collection and structure refinement details are summarized in Table 5 ▸. All hydrogen atoms bound to carbon were geometrically placed and refined using a riding model [C—H = 0.95–1.00 A, *U*_iso_(H) = 1.2 or 1.5 *U*_eq_(C)]. For (I) and (II), the hydrogen atoms of the OH groups were found from difference-Fourier maps and refined as riding atoms in their as-found relative positions with *U*_iso_(H) = 1.5*U*_eq_(O). For (III), the position of the hydrogen atom of the OH group was calculated geometrically (AFIX 147 card in *SHELXL*) and refined using a riding model with *U*_iso_(H) = 1.5*U*_eq_(O). Some reflections blocked by the beam stop were omitted [ten reflections for (I), five reflections for (II) and four reflections for (III)].

## Supplementary Material

Crystal structure: contains datablock(s) I, II, III, global. DOI: 10.1107/S2056989025010709/hb8173sup1.cif

Structure factors: contains datablock(s) I. DOI: 10.1107/S2056989025010709/hb8173Isup2.hkl

Structure factors: contains datablock(s) II. DOI: 10.1107/S2056989025010709/hb8173IIsup3.hkl

Structure factors: contains datablock(s) III. DOI: 10.1107/S2056989025010709/hb8173IIIsup4.hkl

Supporting information file. DOI: 10.1107/S2056989025010709/hb8173Isup5.cml

Supporting information file. DOI: 10.1107/S2056989025010709/hb8173IIsup6.cml

Supporting information file. DOI: 10.1107/S2056989025010709/hb8173IIIsup7.cml

Supplemantary Material. DOI: 10.1107/S2056989025010709/hb8173sup8.pdf

CCDC references: 2512457, 2512456, 2512455

Additional supporting information:  crystallographic information; 3D view; checkCIF report

## Figures and Tables

**Figure 6:**
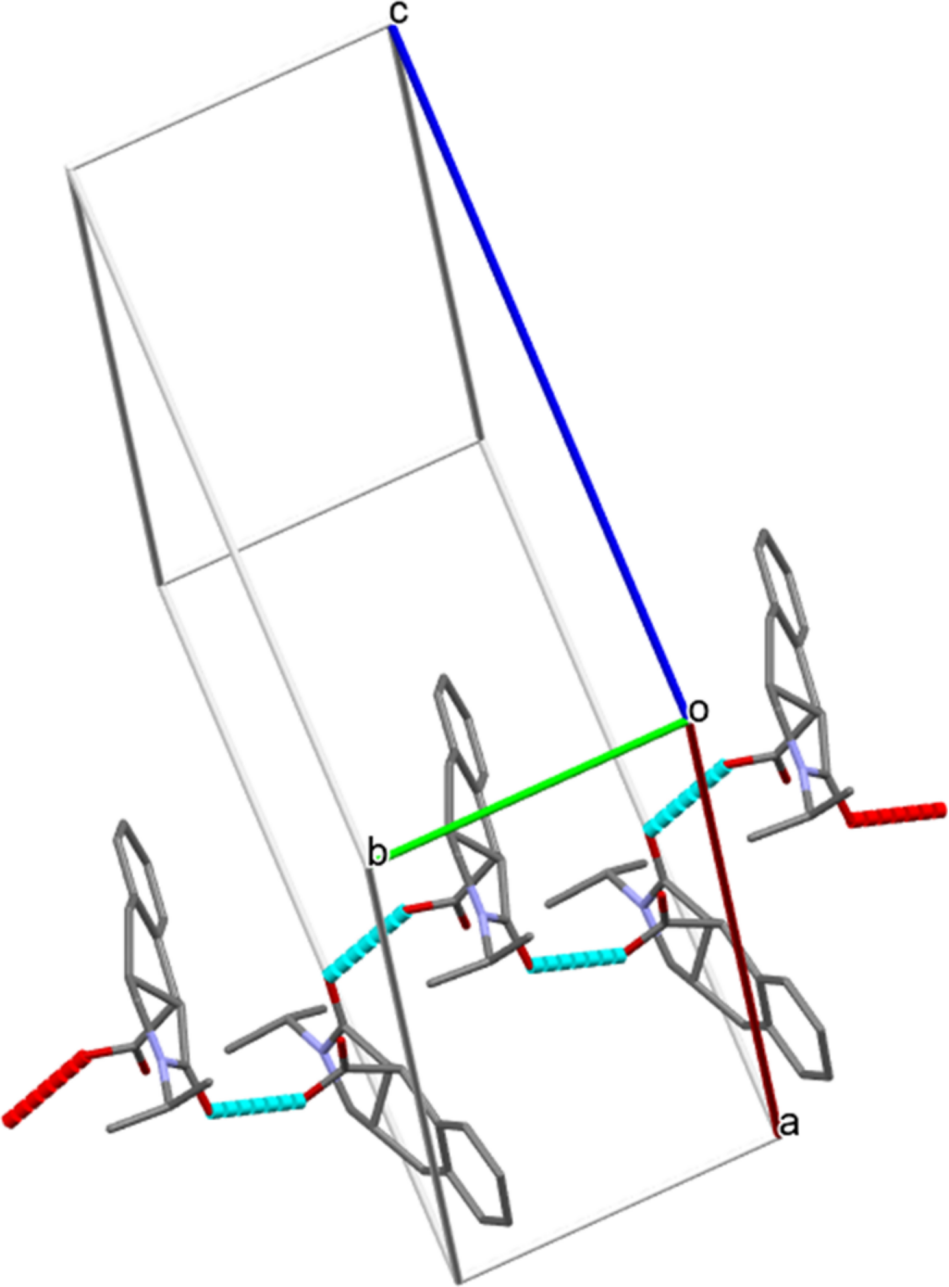
Partial packing diagram for (II) showing O—H⋯O hydrogen bonds forming *C*(7) zigzag chains propagating along the [010] direction. The remaining hydrogen atoms are omitted for clarity.

**Figure 7:**
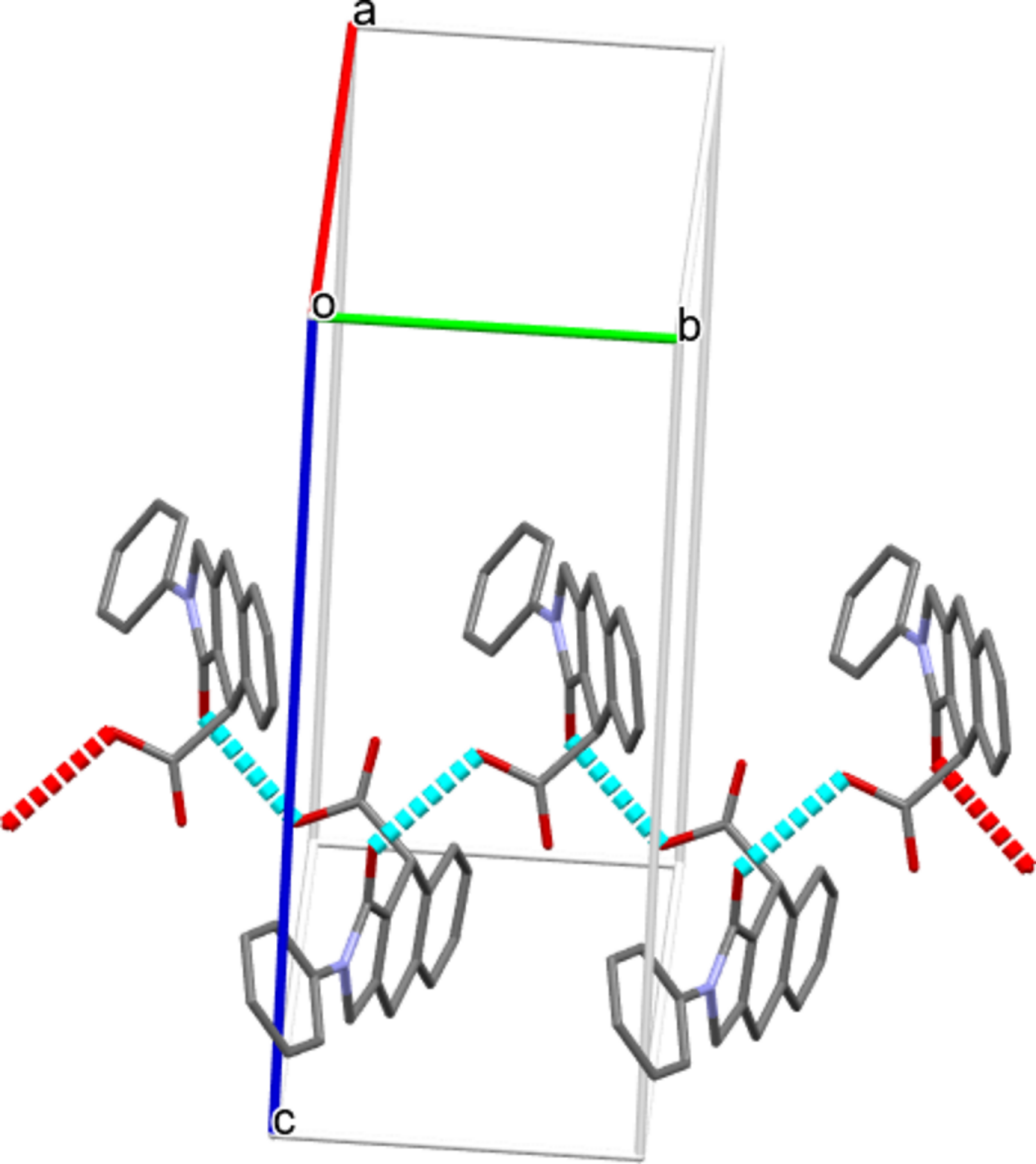
Partial packing diagram for (III) showing O—H⋯O hydrogen bonds forming *C*(7) zigzag chains propagating along the [010] direction. The remaining hydrogen atoms are omitted for clarity.

**Table 1:** Hydrogen-bond geometry (Å, °) for (I) *Cg*3, *Cg*4, *Cg*10 and *Cg*11 are the centroids of the C4–C9, C16–C21, C25–C30 and C37–C42 rings, respectively.

*D*—H⋯*A*	*D*—H	H⋯*A*	*D*⋯*A*	*D*—H⋯*A*
O2—H2*O*⋯O4^i^	0.85	1.77	2.607 (2)	166
O5—H5*O*⋯O1	0.85	1.76	2.606 (2)	171
C2—H2⋯O2	1.00	2.38	3.030 (3)	122
C15—H15*A*⋯O6^i^	0.99	2.52	3.404 (3)	148
C23—H23⋯O5	1.00	2.34	3.018 (3)	124
C3—H3*A*⋯*Cg*3^ii^	0.99	2.75	3.702 (3)	161
C7—H7⋯*Cg*11^iii^	0.95	2.86	3.796 (3)	167
C20—H20⋯*Cg*4^iv^	0.95	2.95	3.783 (3)	148
C24—H24*A*⋯*Cg*10^v^	0.99	2.71	3.648 (3)	158

Symmetry codes: (i) 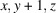; (ii) 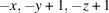; (iii) 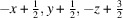; (iv) 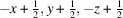; (v) 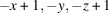.

**Table 2:** Hydrogen-bond geometry (Å, °) for (II) *Cg*3 is the centroid of the C4–C9 rings.

*D*—H⋯*A*	*D*—H	H⋯*A*	*D*⋯*A*	*D*—H⋯*A*
O2—H2*O*⋯O1^i^	0.86	1.74	2.5885 (17)	171
C2—H2⋯O2	1.00	2.33	3.002 (2)	124
C3—H3*B*⋯O2^ii^	0.99	2.56	3.500 (2)	159
C16—H16*A*⋯O3^i^	0.98	2.55	3.531 (3)	176
C3—H3*A*⋯*Cg*3^iii^	0.99	2.68	3.6164 (16)	158

Symmetry codes: (i) 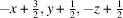; (ii) 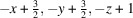; (iii) 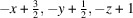.

**Table 3:** Hydrogen-bond geometry (Å, °) for (III) *Cg*1, *Cg*2, *Cg*3 and *Cg*4 are the centroids of the N1/C1/C2/C11/C12, C2–C11 C4–C9 and C16–C21 rings, respectively.

*D*—H⋯*A*	*D*—H	H⋯*A*	*D*⋯*A*	*D*—H⋯*A*
O2—H2*O*⋯O1^i^	0.84	1.90	2.741 (2)	174
C1—H1*A*⋯*Cg*2^ii^	0.99	2.91	3.756 (2)	144
C1—H1*B*⋯*Cg*3^iii^	0.99	2.58	3.427 (2)	144
C3—H3*A*⋯*Cg*1^ii^	0.99	2.70	3.593 (2)	150
C3—H3*B*⋯*Cg*2^iii^	0.99	2.60	3.388 (2)	137
C18—H18⋯*Cg*4^iv^	0.95	2.61	3.452 (2)	148

Symmetry codes: (i) 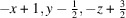; (ii) 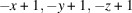; (iii) 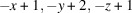; (iv) 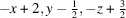.

**Table 4:** The Hirshfeld fingerprint contact percentages (%) for (I), (II) and (III)

Contacts	(I)*A*	(I)*B*	(II)	(III)
H⋯H	53.7	59.8	58.0	39.2
C⋯H/H⋯C	24.7	20.6	14.9	30.6
O⋯H/H⋯O	20.2	18.1	26.7	25.8
O⋯C/C⋯O	1.0	1.1	0.2	0.2
O⋯O	0.3	0.2	0.2	0.3
N⋯H/H⋯N	0.2	0.1	0.0	0.5
N⋯C/C⋯N	0.0	0.0	0.0	0.3
C⋯C	0.0	0.0	0.0	1.7

**Table 5:** Experimental details

	(I)	(II)	(III)
Crystal data			
Chemical formula	C_21_H_21_NO_3_	C_16_H_19_NO_3_	C_19_H_15_NO_3_
*M*_r_	335.39	273.32	305.32
Crystal system, space group	Monoclinic, *P*2_1_/*n*	Monoclinic, *C*2/*c*	Monoclinic, *P*2_1_/*c*
Temperature (K)	150	150	100
*a*, *b*, *c* (Å)	17.3659 (15), 8.0220 (7), 25.883 (2)	25.1799 (12), 7.9085 (3), 17.6079 (8)	11.9915 (10), 7.1296 (6), 16.7967 (16)
β (°)	109.250 (4)	126.771 (2)	99.338 (5)
*V* (Å^3^)	3404.2 (5)	2808.7 (2)	1417.0 (2)
*Z*	8	8	4
Radiation type	Mo *K*α	Mo *K*α	Mo *K*α
μ (mm^−1^)	0.09	0.09	0.10
Crystal size (mm)	0.26 × 0.24 × 0.21	0.31 × 0.29 × 0.29	0.40 × 0.04 × 0.03

Data collection			
Diffractometer	Bruker APEXII CCD	Bruker APEXII CCD	Bruker Kappa APEXII area-detector diffractometer
Absorption correction	Multi-scan (*SADABS*; Krause *et al.*, 2015 ▸)	Multi-scan (*SADABS*; Krause *et al.*, 2015 ▸)	Multi-scan (*SADABS*; Krause *et al.*, 2015 ▸)
*T*_min_, *T*_max_	0.964, 0.973	0.964, 0.967	0.842, 1.000
No. of measured, independent and observed [*I* > 2σ(*I*)] reflections	46007, 6513, 3839	12572, 3240, 2416	13142, 3226, 1938
*R*_int_	0.115	0.036	0.088
(sin θ/λ)_max_ (Å^−1^)	0.612	0.650	0.655

Refinement			
*R*[*F*^2^ > 2σ(*F*^2^)], *wR*(*F*^2^), *S*	0.048, 0.118, 1.01	0.042, 0.108, 1.03	0.054, 0.128, 1.01
No. of reflections	6513	3240	3226
No. of parameters	453	184	209
H-atom treatment	H-atom parameters constrained	H-atom parameters constrained	H-atom parameters constrained
Δρ_max_, Δρ_min_ (e Å^−3^)	0.25, −0.24	0.32, −0.19	0.31, −0.24

Computer programs: *APEX2* (Bruker, 2007 ▸), *APEX3* (Bruker, 2016 ▸), *SAINT* (Bruker, 2007 ▸), *SHELXT* (Sheldrick, 2015*a* ▸), *SHELXL* (Sheldrick, 2015*b* ▸), *ORTEP-3 for Windows* (Farrugia, 2012 ▸) and *PLATON* (Spek, 2020 ▸).

## supplementary crystallographic information

### (3aRS,4RS,9aSR)-3-Oxo-2-(2-phenylethyl)-2,3,3a,4,9,9a-hexahydro-1H-benzo[f]isoindole-4-carboxylic acid (I) Crystal data

**Table:**

C_21_H_21_NO_3_	*F*(000) = 1424
*M**_r_* = 335.39	*D*_x_ = 1.309 Mg m^−^^3^
Monoclinic, *P*2_1_/*n*	Mo *K*α radiation, λ = 0.71073 Å
*a* = 17.3659 (15) Å	Cell parameters from 3824 reflections
*b* = 8.0220 (7) Å	θ = 3.1–20.6°
*c* = 25.883 (2) Å	µ = 0.09 mm^−^^1^
β = 109.250 (4)°	*T* = 150 K
*V* = 3404.2 (5) Å^3^	Block, colourless
*Z* = 8	0.26 × 0.24 × 0.21 mm

### (3aRS,4RS,9aSR)-3-Oxo-2-(2-phenylethyl)-2,3,3a,4,9,9a-hexahydro-1H-benzo[f]isoindole-4-carboxylic acid (I) Data collection

**Table:**

Bruker APEXII CCD diffractometer	3839 reflections with *I* > 2σ(*I*)
φ and ω scans	*R*_int_ = 0.115
Absorption correction: multi-scan (SADABS; Krause *et al.*, 2015)	θ_max_ = 25.8°, θ_min_ = 2.5°
*T*_min_ = 0.964, *T*_max_ = 0.973	*h* = −21→20
46007 measured reflections	*k* = −8→9
6513 independent reflections	*l* = −31→31

### (3aRS,4RS,9aSR)-3-Oxo-2-(2-phenylethyl)-2,3,3a,4,9,9a-hexahydro-1H-benzo[f]isoindole-4-carboxylic acid (I) Refinement

**Table:**

Refinement on *F*^2^	Secondary atom site location: difference Fourier map
Least-squares matrix: full	Hydrogen site location: mixed
*R*[*F*^2^ > 2σ(*F*^2^)] = 0.048	H-atom parameters constrained
*wR*(*F*^2^) = 0.118	*w* = 1/[σ^2^(*F*_o_^2^) + (0.0479*P*)^2^ + 0.106*P*] where *P* = (*F*_o_^2^ + 2*F*_c_^2^)/3
*S* = 1.01	(Δ/σ)_max_ < 0.001
6513 reflections	Δρ_max_ = 0.25 e Å^−^^3^
453 parameters	Δρ_min_ = −0.24 e Å^−^^3^
0 restraints	Extinction correction: SHELXL-2019/2 (Sheldrick, 2008), Fc^*^=kFc[1+0.001xFc^2^λ^3^/sin(2θ)]^-1/4^
Primary atom site location: difference Fourier map	Extinction coefficient: 0.0022 (4)

### (3aRS,4RS,9aSR)-3-Oxo-2-(2-phenylethyl)-2,3,3a,4,9,9a-hexahydro-1H-benzo[f]isoindole-4-carboxylic acid (I) Special details

**Table:**

Geometry. All esds (except the esd in the dihedral angle between two l.s. planes) are estimated using the full covariance matrix. The cell esds are taken into account individually in the estimation of esds in distances, angles and torsion angles; correlations between esds in cell parameters are only used when they are defined by crystal symmetry. An approximate (isotropic) treatment of cell esds is used for estimating esds involving l.s. planes.

### (3aRS,4RS,9aSR)-3-Oxo-2-(2-phenylethyl)-2,3,3a,4,9,9a-hexahydro-1H-benzo[f]isoindole-4-carboxylic acid (I) Fractional atomic coordinates and isotropic or equivalent isotropic displacement parameters (Å^2^)

**Table:**

	*x*	*y*	*z*	*U*_iso_*/*U*_eq_	
C1	0.00531 (13)	0.8263 (3)	0.37497 (9)	0.0267 (6)	
H1A	−0.027011	0.727334	0.357639	0.032*	
H1B	−0.025994	0.928424	0.359831	0.032*	
C2	0.02821 (13)	0.8200 (3)	0.43681 (9)	0.0225 (5)	
H2	0.040733	0.936136	0.451174	0.027*	
C3	−0.02924 (14)	0.7432 (3)	0.46274 (10)	0.0256 (6)	
H3A	−0.048201	0.633629	0.445696	0.031*	
H3B	−0.077502	0.815969	0.456545	0.031*	
C4	0.01362 (13)	0.7211 (3)	0.52363 (10)	0.0228 (5)	
C5	−0.03361 (14)	0.7005 (2)	0.55757 (10)	0.0243 (6)	
H5	−0.091370	0.702577	0.541975	0.029*	
C6	0.00147 (15)	0.6773 (3)	0.61309 (10)	0.0302 (6)	
H6	−0.032011	0.660899	0.635202	0.036*	
C7	0.08581 (15)	0.6778 (3)	0.63680 (10)	0.0299 (6)	
H7	0.110444	0.663129	0.675156	0.036*	
C8	0.13333 (15)	0.6999 (3)	0.60377 (10)	0.0267 (6)	
H8	0.191000	0.702338	0.619989	0.032*	
C9	0.09887 (14)	0.7186 (3)	0.54735 (9)	0.0212 (5)	
C10	0.15700 (13)	0.7315 (3)	0.51393 (9)	0.0207 (5)	
H10	0.193934	0.632332	0.523065	0.025*	
C11	0.10774 (13)	0.7221 (3)	0.45326 (9)	0.0199 (5)	
H11	0.092238	0.602434	0.445372	0.024*	
C12	0.14558 (14)	0.7756 (3)	0.41154 (10)	0.0228 (5)	
C13	0.20944 (14)	0.8854 (3)	0.53104 (10)	0.0224 (5)	
C14	0.09812 (15)	0.8727 (3)	0.31641 (9)	0.0272 (6)	
H14A	0.051839	0.831791	0.285140	0.033*	
H14B	0.148479	0.818717	0.314697	0.033*	
C15	0.10546 (16)	1.0592 (3)	0.31188 (10)	0.0319 (6)	
H15A	0.145507	1.100805	0.346298	0.038*	
H15B	0.052094	1.110252	0.308441	0.038*	
C16	0.13121 (13)	1.1178 (3)	0.26455 (9)	0.0254 (6)	
C17	0.12527 (14)	1.0192 (3)	0.21935 (10)	0.0308 (6)	
H17	0.103962	0.909440	0.217360	0.037*	
C18	0.15000 (15)	1.0788 (3)	0.17710 (10)	0.0358 (7)	
H18	0.145687	1.009313	0.146546	0.043*	
C19	0.18044 (15)	1.2360 (3)	0.17893 (10)	0.0368 (7)	
H19	0.196701	1.276584	0.149623	0.044*	
C20	0.18753 (15)	1.3364 (3)	0.22390 (11)	0.0367 (7)	
H20	0.209018	1.445889	0.225569	0.044*	
C21	0.16339 (14)	1.2772 (3)	0.26620 (10)	0.0324 (6)	
H21	0.168847	1.346437	0.296974	0.039*	
N1	0.08530 (11)	0.8272 (2)	0.36734 (8)	0.0252 (5)	
O1	0.21861 (9)	0.76834 (18)	0.41499 (6)	0.0271 (4)	
O3	0.27549 (10)	0.8855 (2)	0.56610 (7)	0.0366 (4)	
O2	0.17441 (9)	1.02119 (18)	0.50437 (6)	0.0284 (4)	
H2O	0.199197	1.104154	0.522907	0.043*	
C22	0.45186 (13)	0.3870 (3)	0.60586 (9)	0.0227 (5)	
H22A	0.480811	0.298001	0.631388	0.027*	
H22B	0.480042	0.494575	0.617795	0.027*	
C23	0.44539 (13)	0.3463 (3)	0.54738 (9)	0.0194 (5)	
H23	0.433400	0.452307	0.525853	0.023*	
C24	0.51382 (13)	0.2595 (3)	0.53475 (9)	0.0218 (5)	
H24A	0.529278	0.156929	0.557025	0.026*	
H24B	0.562126	0.333408	0.543784	0.026*	
C25	0.48595 (13)	0.2158 (2)	0.47455 (9)	0.0203 (5)	
C26	0.54492 (14)	0.1906 (2)	0.44950 (10)	0.0236 (5)	
H26	0.600898	0.203525	0.470471	0.028*	
C27	0.52385 (14)	0.1472 (3)	0.39495 (10)	0.0263 (6)	
H27	0.565010	0.129243	0.378825	0.032*	
C28	0.44271 (15)	0.1301 (3)	0.36404 (10)	0.0291 (6)	
H28	0.427572	0.100903	0.326439	0.035*	
C29	0.38336 (14)	0.1557 (3)	0.38823 (10)	0.0257 (6)	
H29	0.327542	0.143902	0.366743	0.031*	
C30	0.40352 (13)	0.1983 (2)	0.44307 (9)	0.0201 (5)	
C31	0.33357 (13)	0.2208 (3)	0.46654 (9)	0.0203 (5)	
H31	0.300024	0.116756	0.458484	0.024*	
C32	0.36894 (13)	0.2386 (3)	0.52879 (9)	0.0192 (5)	
H32	0.384742	0.124478	0.544220	0.023*	
C33	0.31636 (14)	0.3142 (2)	0.55860 (9)	0.0195 (5)	
C34	0.27825 (14)	0.3646 (3)	0.43917 (9)	0.0210 (5)	
C35	0.33808 (15)	0.4828 (3)	0.64180 (10)	0.0282 (6)	
H35A	0.278265	0.469746	0.631919	0.034*	
H35B	0.350019	0.603238	0.640875	0.034*	
C36	0.37905 (15)	0.4170 (3)	0.69935 (10)	0.0313 (6)	
H36A	0.438734	0.432398	0.709271	0.038*	
H36B	0.360449	0.483667	0.725182	0.038*	
C37	0.36169 (16)	0.2360 (3)	0.70593 (10)	0.0300 (6)	
C38	0.28263 (17)	0.1814 (3)	0.69700 (11)	0.0409 (7)	
H38	0.239231	0.259835	0.686347	0.049*	
C39	0.2653 (2)	0.0168 (4)	0.70315 (13)	0.0569 (9)	
H39	0.210617	−0.017631	0.696692	0.068*	
C40	0.3272 (3)	−0.0968 (4)	0.71858 (13)	0.0648 (10)	
H40	0.315728	−0.210446	0.723445	0.078*	
C41	0.4059 (2)	−0.0468 (4)	0.72706 (12)	0.0616 (10)	
H41	0.448742	−0.126514	0.737272	0.074*	
C42	0.42356 (18)	0.1196 (3)	0.72083 (10)	0.0434 (7)	
H42	0.478296	0.153178	0.726845	0.052*	
N2	0.36555 (11)	0.3963 (2)	0.60185 (7)	0.0213 (4)	
O4	0.24114 (9)	0.30285 (17)	0.54630 (6)	0.0256 (4)	
O5	0.30897 (9)	0.51159 (18)	0.45799 (6)	0.0284 (4)	
H5O	0.281478	0.600091	0.447130	0.043*	
O6	0.21283 (10)	0.3465 (2)	0.40449 (7)	0.0380 (5)	

### (3aRS,4RS,9aSR)-3-Oxo-2-(2-phenylethyl)-2,3,3a,4,9,9a-hexahydro-1H-benzo[f]isoindole-4-carboxylic acid (I) Atomic displacement parameters (Å^2^)

**Table:**

	*U*^11^	*U*^22^	*U*^33^	*U*^12^	*U*^13^	*U*^23^
C1	0.0245 (13)	0.0277 (13)	0.0275 (14)	−0.0016 (11)	0.0079 (11)	0.0041 (11)
C2	0.0208 (13)	0.0213 (13)	0.0268 (14)	0.0013 (10)	0.0099 (11)	0.0034 (10)
C3	0.0219 (13)	0.0220 (13)	0.0346 (15)	0.0023 (10)	0.0116 (12)	0.0039 (11)
C4	0.0245 (13)	0.0135 (12)	0.0322 (15)	0.0003 (10)	0.0117 (12)	0.0037 (10)
C5	0.0268 (13)	0.0139 (12)	0.0380 (16)	0.0012 (10)	0.0187 (12)	0.0031 (11)
C6	0.0418 (17)	0.0205 (13)	0.0406 (17)	−0.0008 (11)	0.0302 (14)	0.0003 (11)
C7	0.0417 (16)	0.0291 (14)	0.0223 (14)	−0.0005 (12)	0.0151 (13)	−0.0025 (11)
C8	0.0286 (14)	0.0265 (13)	0.0282 (15)	0.0013 (11)	0.0134 (12)	−0.0001 (11)
C9	0.0266 (13)	0.0147 (12)	0.0272 (14)	0.0007 (10)	0.0154 (11)	0.0010 (10)
C10	0.0220 (13)	0.0186 (12)	0.0251 (14)	0.0037 (10)	0.0125 (11)	0.0044 (10)
C11	0.0215 (13)	0.0147 (12)	0.0256 (14)	−0.0023 (9)	0.0108 (11)	0.0005 (10)
C12	0.0308 (15)	0.0138 (12)	0.0273 (14)	0.0039 (10)	0.0146 (12)	0.0021 (10)
C13	0.0188 (13)	0.0267 (14)	0.0255 (14)	0.0044 (11)	0.0126 (12)	0.0027 (11)
C14	0.0325 (14)	0.0290 (14)	0.0207 (14)	0.0024 (11)	0.0096 (12)	0.0028 (11)
C15	0.0443 (16)	0.0298 (14)	0.0254 (15)	−0.0031 (12)	0.0167 (13)	−0.0005 (11)
C16	0.0240 (13)	0.0309 (14)	0.0210 (14)	0.0013 (11)	0.0072 (11)	0.0023 (11)
C17	0.0331 (15)	0.0332 (15)	0.0247 (15)	0.0020 (11)	0.0074 (12)	0.0019 (12)
C18	0.0349 (16)	0.0507 (18)	0.0221 (15)	0.0075 (13)	0.0100 (13)	0.0025 (12)
C19	0.0247 (14)	0.0597 (19)	0.0264 (16)	0.0015 (13)	0.0090 (12)	0.0148 (14)
C20	0.0295 (15)	0.0429 (17)	0.0347 (17)	−0.0076 (12)	0.0066 (13)	0.0137 (13)
C21	0.0316 (15)	0.0362 (15)	0.0266 (15)	−0.0045 (12)	0.0057 (12)	0.0007 (12)
N1	0.0237 (11)	0.0283 (11)	0.0248 (11)	0.0027 (9)	0.0096 (9)	0.0066 (9)
O1	0.0259 (10)	0.0283 (9)	0.0329 (10)	0.0077 (7)	0.0175 (8)	0.0077 (7)
O3	0.0230 (10)	0.0379 (10)	0.0423 (12)	−0.0019 (8)	0.0017 (9)	0.0059 (9)
O2	0.0243 (9)	0.0184 (9)	0.0390 (11)	−0.0010 (7)	0.0057 (8)	−0.0008 (7)
C22	0.0245 (13)	0.0207 (12)	0.0223 (13)	−0.0016 (10)	0.0070 (11)	−0.0011 (10)
C23	0.0219 (12)	0.0176 (12)	0.0194 (13)	−0.0016 (10)	0.0078 (10)	0.0003 (10)
C24	0.0200 (13)	0.0207 (12)	0.0242 (13)	−0.0023 (10)	0.0068 (11)	0.0004 (10)
C25	0.0238 (13)	0.0133 (12)	0.0260 (14)	0.0002 (9)	0.0112 (11)	0.0013 (10)
C26	0.0237 (13)	0.0169 (12)	0.0335 (15)	0.0008 (10)	0.0140 (12)	0.0033 (11)
C27	0.0314 (15)	0.0210 (13)	0.0338 (15)	0.0037 (11)	0.0205 (13)	0.0020 (11)
C28	0.0408 (16)	0.0261 (14)	0.0232 (14)	0.0040 (12)	0.0143 (13)	−0.0017 (11)
C29	0.0271 (14)	0.0242 (13)	0.0252 (14)	0.0019 (11)	0.0079 (11)	−0.0026 (11)
C30	0.0245 (13)	0.0126 (11)	0.0253 (13)	0.0014 (10)	0.0110 (11)	−0.0003 (10)
C31	0.0214 (12)	0.0180 (12)	0.0214 (13)	−0.0017 (10)	0.0072 (10)	−0.0016 (10)
C32	0.0227 (13)	0.0156 (11)	0.0204 (13)	0.0000 (9)	0.0086 (11)	0.0006 (9)
C33	0.0255 (14)	0.0119 (11)	0.0230 (13)	−0.0011 (10)	0.0102 (11)	0.0026 (10)
C34	0.0192 (13)	0.0256 (14)	0.0211 (13)	−0.0002 (10)	0.0105 (11)	−0.0019 (10)
C35	0.0371 (15)	0.0217 (13)	0.0301 (15)	0.0014 (11)	0.0168 (12)	−0.0037 (11)
C36	0.0404 (16)	0.0319 (15)	0.0250 (15)	−0.0015 (12)	0.0155 (13)	−0.0055 (11)
C37	0.0426 (16)	0.0306 (14)	0.0217 (14)	0.0048 (12)	0.0171 (12)	−0.0014 (11)
C38	0.0528 (19)	0.0344 (16)	0.0426 (18)	−0.0021 (14)	0.0251 (15)	−0.0026 (13)
C39	0.085 (2)	0.0398 (19)	0.062 (2)	−0.0143 (18)	0.047 (2)	−0.0062 (16)
C40	0.133 (3)	0.0323 (18)	0.055 (2)	−0.002 (2)	0.067 (2)	0.0016 (16)
C41	0.109 (3)	0.043 (2)	0.048 (2)	0.035 (2)	0.047 (2)	0.0138 (15)
C42	0.0569 (19)	0.0497 (18)	0.0289 (16)	0.0168 (15)	0.0214 (14)	0.0085 (13)
N2	0.0226 (11)	0.0223 (10)	0.0209 (11)	−0.0001 (8)	0.0095 (9)	−0.0025 (8)
O4	0.0196 (9)	0.0242 (9)	0.0352 (10)	−0.0025 (7)	0.0121 (8)	−0.0029 (7)
O5	0.0245 (9)	0.0158 (9)	0.0382 (11)	0.0010 (7)	0.0015 (8)	0.0014 (7)
O6	0.0287 (10)	0.0371 (11)	0.0366 (11)	0.0006 (8)	−0.0050 (9)	−0.0044 (8)

### (3aRS,4RS,9aSR)-3-Oxo-2-(2-phenylethyl)-2,3,3a,4,9,9a-hexahydro-1H-benzo[f]isoindole-4-carboxylic acid (I) Geometric parameters (Å, º)

**Table:**

C1—N1	1.466 (3)	C22—N2	1.470 (3)
C1—C2	1.518 (3)	C22—C23	1.516 (3)
C1—H1A	0.9900	C22—H22A	0.9900
C1—H1B	0.9900	C22—H22B	0.9900
C2—C3	1.505 (3)	C23—C24	1.503 (3)
C2—C11	1.522 (3)	C23—C32	1.523 (3)
C2—H2	1.0000	C23—H23	1.0000
C3—C4	1.515 (3)	C24—C25	1.513 (3)
C3—H3A	0.9900	C24—H24A	0.9900
C3—H3B	0.9900	C24—H24B	0.9900
C4—C5	1.395 (3)	C25—C26	1.396 (3)
C4—C9	1.404 (3)	C25—C30	1.401 (3)
C5—C6	1.376 (3)	C26—C27	1.381 (3)
C5—H5	0.9500	C26—H26	0.9500
C6—C7	1.389 (3)	C27—C28	1.379 (3)
C6—H6	0.9500	C27—H27	0.9500
C7—C8	1.381 (3)	C28—C29	1.386 (3)
C7—H7	0.9500	C28—H28	0.9500
C8—C9	1.392 (3)	C29—C30	1.388 (3)
C8—H8	0.9500	C29—H29	0.9500
C9—C10	1.534 (3)	C30—C31	1.538 (3)
C10—C13	1.511 (3)	C31—C34	1.518 (3)
C10—C11	1.522 (3)	C31—C32	1.530 (3)
C10—H10	1.0000	C31—H31	1.0000
C11—C12	1.500 (3)	C32—C33	1.504 (3)
C11—H11	1.0000	C32—H32	1.0000
C12—O1	1.243 (3)	C33—O4	1.242 (2)
C12—N1	1.336 (3)	C33—N2	1.336 (3)
C13—O3	1.205 (3)	C34—O6	1.202 (3)
C13—O2	1.325 (3)	C34—O5	1.320 (3)
C14—N1	1.454 (3)	C35—N2	1.450 (3)
C14—C15	1.509 (3)	C35—C36	1.518 (3)
C14—H14A	0.9900	C35—H35A	0.9900
C14—H14B	0.9900	C35—H35B	0.9900
C15—C16	1.511 (3)	C36—C37	1.504 (3)
C15—H15A	0.9900	C36—H36A	0.9900
C15—H15B	0.9900	C36—H36B	0.9900
C16—C17	1.388 (3)	C37—C42	1.379 (3)
C16—C21	1.390 (3)	C37—C38	1.386 (3)
C17—C18	1.385 (3)	C38—C39	1.375 (4)
C17—H17	0.9500	C38—H38	0.9500
C18—C19	1.362 (4)	C39—C40	1.364 (4)
C18—H18	0.9500	C39—H39	0.9500
C19—C20	1.387 (4)	C40—C41	1.371 (5)
C19—H19	0.9500	C40—H40	0.9500
C20—C21	1.379 (3)	C41—C42	1.391 (4)
C20—H20	0.9500	C41—H41	0.9500
C21—H21	0.9500	C42—H42	0.9500
O2—H2O	0.8498	O5—H5O	0.8500
			
N1—C1—C2	102.23 (17)	N2—C22—C23	101.68 (17)
N1—C1—H1A	111.3	N2—C22—H22A	111.4
C2—C1—H1A	111.3	C23—C22—H22A	111.4
N1—C1—H1B	111.3	N2—C22—H22B	111.4
C2—C1—H1B	111.3	C23—C22—H22B	111.4
H1A—C1—H1B	109.2	H22A—C22—H22B	109.3
C3—C2—C1	120.13 (19)	C24—C23—C22	120.88 (19)
C3—C2—C11	109.75 (18)	C24—C23—C32	109.73 (17)
C1—C2—C11	102.13 (17)	C22—C23—C32	101.97 (17)
C3—C2—H2	108.1	C24—C23—H23	107.9
C1—C2—H2	108.1	C22—C23—H23	107.9
C11—C2—H2	108.1	C32—C23—H23	107.9
C2—C3—C4	109.94 (19)	C23—C24—C25	108.91 (18)
C2—C3—H3A	109.7	C23—C24—H24A	109.9
C4—C3—H3A	109.7	C25—C24—H24A	109.9
C2—C3—H3B	109.7	C23—C24—H24B	109.9
C4—C3—H3B	109.7	C25—C24—H24B	109.9
H3A—C3—H3B	108.2	H24A—C24—H24B	108.3
C5—C4—C9	118.5 (2)	C26—C25—C30	118.7 (2)
C5—C4—C3	118.7 (2)	C26—C25—C24	118.5 (2)
C9—C4—C3	122.8 (2)	C30—C25—C24	122.73 (19)
C6—C5—C4	121.6 (2)	C27—C26—C25	121.6 (2)
C6—C5—H5	119.2	C27—C26—H26	119.2
C4—C5—H5	119.2	C25—C26—H26	119.2
C5—C6—C7	120.0 (2)	C28—C27—C26	119.6 (2)
C5—C6—H6	120.0	C28—C27—H27	120.2
C7—C6—H6	120.0	C26—C27—H27	120.2
C8—C7—C6	119.0 (2)	C27—C28—C29	119.5 (2)
C8—C7—H7	120.5	C27—C28—H28	120.2
C6—C7—H7	120.5	C29—C28—H28	120.2
C7—C8—C9	121.7 (2)	C28—C29—C30	121.6 (2)
C7—C8—H8	119.2	C28—C29—H29	119.2
C9—C8—H8	119.2	C30—C29—H29	119.2
C8—C9—C4	119.2 (2)	C29—C30—C25	119.0 (2)
C8—C9—C10	117.6 (2)	C29—C30—C31	117.9 (2)
C4—C9—C10	123.2 (2)	C25—C30—C31	123.2 (2)
C13—C10—C11	114.95 (18)	C34—C31—C32	113.18 (17)
C13—C10—C9	109.55 (17)	C34—C31—C30	111.44 (17)
C11—C10—C9	109.14 (18)	C32—C31—C30	109.38 (18)
C13—C10—H10	107.6	C34—C31—H31	107.5
C11—C10—H10	107.6	C32—C31—H31	107.5
C9—C10—H10	107.6	C30—C31—H31	107.5
C12—C11—C2	102.93 (18)	C33—C32—C23	102.84 (17)
C12—C11—C10	119.87 (19)	C33—C32—C31	118.48 (18)
C2—C11—C10	114.36 (18)	C23—C32—C31	113.01 (17)
C12—C11—H11	106.2	C33—C32—H32	107.3
C2—C11—H11	106.2	C23—C32—H32	107.3
C10—C11—H11	106.2	C31—C32—H32	107.3
O1—C12—N1	124.6 (2)	O4—C33—N2	125.4 (2)
O1—C12—C11	127.9 (2)	O4—C33—C32	127.2 (2)
N1—C12—C11	107.42 (19)	N2—C33—C32	107.46 (18)
O3—C13—O2	123.5 (2)	O6—C34—O5	123.4 (2)
O3—C13—C10	123.6 (2)	O6—C34—C31	123.6 (2)
O2—C13—C10	113.0 (2)	O5—C34—C31	112.94 (19)
N1—C14—C15	111.21 (18)	N2—C35—C36	112.03 (19)
N1—C14—H14A	109.4	N2—C35—H35A	109.2
C15—C14—H14A	109.4	C36—C35—H35A	109.2
N1—C14—H14B	109.4	N2—C35—H35B	109.2
C15—C14—H14B	109.4	C36—C35—H35B	109.2
H14A—C14—H14B	108.0	H35A—C35—H35B	107.9
C14—C15—C16	115.26 (19)	C37—C36—C35	113.6 (2)
C14—C15—H15A	108.5	C37—C36—H36A	108.8
C16—C15—H15A	108.5	C35—C36—H36A	108.8
C14—C15—H15B	108.5	C37—C36—H36B	108.8
C16—C15—H15B	108.5	C35—C36—H36B	108.8
H15A—C15—H15B	107.5	H36A—C36—H36B	107.7
C17—C16—C21	117.9 (2)	C42—C37—C38	118.1 (2)
C17—C16—C15	123.1 (2)	C42—C37—C36	121.2 (2)
C21—C16—C15	119.0 (2)	C38—C37—C36	120.7 (2)
C18—C17—C16	120.8 (2)	C39—C38—C37	121.7 (3)
C18—C17—H17	119.6	C39—C38—H38	119.1
C16—C17—H17	119.6	C37—C38—H38	119.1
C19—C18—C17	120.6 (2)	C40—C39—C38	119.7 (3)
C19—C18—H18	119.7	C40—C39—H39	120.2
C17—C18—H18	119.7	C38—C39—H39	120.2
C18—C19—C20	119.5 (2)	C39—C40—C41	119.9 (3)
C18—C19—H19	120.2	C39—C40—H40	120.1
C20—C19—H19	120.2	C41—C40—H40	120.1
C21—C20—C19	120.0 (2)	C40—C41—C42	120.6 (3)
C21—C20—H20	120.0	C40—C41—H41	119.7
C19—C20—H20	120.0	C42—C41—H41	119.7
C20—C21—C16	121.1 (2)	C37—C42—C41	120.0 (3)
C20—C21—H21	119.4	C37—C42—H42	120.0
C16—C21—H21	119.4	C41—C42—H42	120.0
C12—N1—C14	122.89 (19)	C33—N2—C35	124.28 (19)
C12—N1—C1	113.12 (18)	C33—N2—C22	112.91 (17)
C14—N1—C1	123.94 (18)	C35—N2—C22	122.79 (18)
C13—O2—H2O	106.8	C34—O5—H5O	120.7
			
N1—C1—C2—C3	−152.87 (19)	N2—C22—C23—C24	−155.55 (18)
N1—C1—C2—C11	−31.3 (2)	N2—C22—C23—C32	−33.6 (2)
C1—C2—C3—C4	168.90 (19)	C22—C23—C24—C25	172.71 (18)
C11—C2—C3—C4	51.1 (2)	C32—C23—C24—C25	54.6 (2)
C2—C3—C4—C5	160.80 (19)	C23—C24—C25—C26	157.38 (18)
C2—C3—C4—C9	−19.9 (3)	C23—C24—C25—C30	−23.2 (3)
C9—C4—C5—C6	−0.3 (3)	C30—C25—C26—C27	−0.7 (3)
C3—C4—C5—C6	179.0 (2)	C24—C25—C26—C27	178.7 (2)
C4—C5—C6—C7	1.5 (3)	C25—C26—C27—C28	0.8 (3)
C5—C6—C7—C8	−0.7 (3)	C26—C27—C28—C29	−0.4 (3)
C6—C7—C8—C9	−1.2 (3)	C27—C28—C29—C30	−0.1 (3)
C7—C8—C9—C4	2.3 (3)	C28—C29—C30—C25	0.1 (3)
C7—C8—C9—C10	−176.3 (2)	C28—C29—C30—C31	−178.80 (19)
C5—C4—C9—C8	−1.5 (3)	C26—C25—C30—C29	0.3 (3)
C3—C4—C9—C8	179.2 (2)	C24—C25—C30—C29	−179.13 (19)
C5—C4—C9—C10	176.96 (19)	C26—C25—C30—C31	179.13 (18)
C3—C4—C9—C10	−2.3 (3)	C24—C25—C30—C31	−0.3 (3)
C8—C9—C10—C13	−62.7 (2)	C29—C30—C31—C34	−63.4 (2)
C4—C9—C10—C13	118.8 (2)	C25—C30—C31—C34	117.8 (2)
C8—C9—C10—C11	170.67 (18)	C29—C30—C31—C32	170.71 (18)
C4—C9—C10—C11	−7.9 (3)	C25—C30—C31—C32	−8.1 (3)
C3—C2—C11—C12	162.37 (18)	C24—C23—C32—C33	163.64 (17)
C1—C2—C11—C12	33.9 (2)	C22—C23—C32—C33	34.3 (2)
C3—C2—C11—C10	−66.0 (2)	C24—C23—C32—C31	−67.5 (2)
C1—C2—C11—C10	165.54 (18)	C22—C23—C32—C31	163.25 (17)
C13—C10—C11—C12	40.6 (3)	C34—C31—C32—C33	36.2 (3)
C9—C10—C11—C12	164.08 (18)	C30—C31—C32—C33	161.06 (17)
C13—C10—C11—C2	−82.3 (2)	C34—C31—C32—C23	−84.2 (2)
C9—C10—C11—C2	41.2 (2)	C30—C31—C32—C23	40.7 (2)
C2—C11—C12—O1	158.7 (2)	C23—C32—C33—O4	158.3 (2)
C10—C11—C12—O1	30.4 (3)	C31—C32—C33—O4	32.9 (3)
C2—C11—C12—N1	−24.2 (2)	C23—C32—C33—N2	−22.3 (2)
C10—C11—C12—N1	−152.48 (19)	C31—C32—C33—N2	−147.68 (19)
C11—C10—C13—O3	−144.5 (2)	C32—C31—C34—O6	−134.0 (2)
C9—C10—C13—O3	92.2 (3)	C30—C31—C34—O6	102.2 (2)
C11—C10—C13—O2	37.0 (3)	C32—C31—C34—O5	45.0 (2)
C9—C10—C13—O2	−86.3 (2)	C30—C31—C34—O5	−78.7 (2)
N1—C14—C15—C16	171.36 (19)	N2—C35—C36—C37	−61.4 (3)
C14—C15—C16—C17	18.7 (3)	C35—C36—C37—C42	118.4 (3)
C14—C15—C16—C21	−160.5 (2)	C35—C36—C37—C38	−61.5 (3)
C21—C16—C17—C18	−0.6 (4)	C42—C37—C38—C39	0.6 (4)
C15—C16—C17—C18	−179.8 (2)	C36—C37—C38—C39	−179.5 (2)
C16—C17—C18—C19	−0.3 (4)	C37—C38—C39—C40	0.2 (4)
C17—C18—C19—C20	0.7 (4)	C38—C39—C40—C41	−1.0 (5)
C18—C19—C20—C21	−0.4 (4)	C39—C40—C41—C42	0.9 (5)
C19—C20—C21—C16	−0.5 (4)	C38—C37—C42—C41	−0.7 (4)
C17—C16—C21—C20	1.0 (4)	C36—C37—C42—C41	179.5 (2)
C15—C16—C21—C20	−179.8 (2)	C40—C41—C42—C37	−0.1 (4)
O1—C12—N1—C14	3.5 (3)	O4—C33—N2—C35	1.1 (3)
C11—C12—N1—C14	−173.70 (18)	C32—C33—N2—C35	−178.29 (18)
O1—C12—N1—C1	−178.8 (2)	O4—C33—N2—C22	179.74 (19)
C11—C12—N1—C1	4.0 (2)	C32—C33—N2—C22	0.3 (2)
C15—C14—N1—C12	−98.4 (2)	C36—C35—N2—C33	121.7 (2)
C15—C14—N1—C1	84.2 (3)	C36—C35—N2—C22	−56.8 (3)
C2—C1—N1—C12	17.9 (2)	C23—C22—N2—C33	21.8 (2)
C2—C1—N1—C14	−164.43 (19)	C23—C22—N2—C35	−159.60 (18)

### (3aRS,4RS,9aSR)-3-Oxo-2-(2-phenylethyl)-2,3,3a,4,9,9a-hexahydro-1H-benzo[f]isoindole-4-carboxylic acid (I) Hydrogen-bond geometry (Å, º)

*Cg*3, *Cg*4, *Cg*10 and *Cg*11 are the centroids of the C4–C9, C16–C21, C25–C30 and C37–C42 rings, respectively.

**Table:**

*D*—H···*A*	*D*—H	H···*A*	*D*···*A*	*D*—H···*A*
O2—H2*O*···O4^i^	0.85	1.77	2.607 (2)	166
O5—H5*O*···O1	0.85	1.76	2.606 (2)	171
C2—H2···O2	1.00	2.38	3.030 (3)	122
C15—H15*A*···O6^i^	0.99	2.52	3.404 (3)	148
C23—H23···O5	1.00	2.34	3.018 (3)	124
C3—H3*A*···*Cg*3^ii^	0.99	2.75	3.702 (3)	161
C7—H7···*Cg*11^iii^	0.95	2.86	3.796 (3)	167
C20—H20···*Cg*4^iv^	0.95	2.95	3.783 (3)	148
C24—H24*A*···*Cg*10^v^	0.99	2.71	3.648 (3)	158

Symmetry codes: (i) *x*, *y*+1, *z*; (ii) −*x*, −*y*+1, −*z*+1; (iii) −*x*+1/2, *y*+1/2, −*z*+3/2; (iv) −*x*+1/2, *y*+1/2, −*z*+1/2; (v) −*x*+1, −*y*, −*z*+1.

### (3aRS,4RS,9aSR)-3-Oxo-2-(propan-2-yl)-2,3,3a,4,9,9a-hexahydro-1H-benzo[f]isoindole-4-carboxylic acid (II) Crystal data

**Table:**

C_16_H_19_NO_3_	*F*(000) = 1168
*M**_r_* = 273.32	*D*_x_ = 1.293 Mg m^−^^3^
Monoclinic, *C*2/*c*	Mo *K*α radiation, λ = 0.71073 Å
*a* = 25.1799 (12) Å	Cell parameters from 2994 reflections
*b* = 7.9085 (3) Å	θ = 2.8–25.0°
*c* = 17.6079 (8) Å	µ = 0.09 mm^−^^1^
β = 126.771 (2)°	*T* = 150 K
*V* = 2808.7 (2) Å^3^	Block, colourless
*Z* = 8	0.31 × 0.29 × 0.29 mm

### (3aRS,4RS,9aSR)-3-Oxo-2-(propan-2-yl)-2,3,3a,4,9,9a-hexahydro-1H-benzo[f]isoindole-4-carboxylic acid (II) Data collection

**Table:**

Bruker APEXII CCD diffractometer	2416 reflections with *I* > 2σ(*I*)
φ and ω scans	*R*_int_ = 0.036
Absorption correction: multi-scan (SADABS; Krause *et al.*, 2015)	θ_max_ = 27.5°, θ_min_ = 3.3°
*T*_min_ = 0.964, *T*_max_ = 0.967	*h* = −32→31
12572 measured reflections	*k* = −10→10
3240 independent reflections	*l* = −22→22

### (3aRS,4RS,9aSR)-3-Oxo-2-(propan-2-yl)-2,3,3a,4,9,9a-hexahydro-1H-benzo[f]isoindole-4-carboxylic acid (II) Refinement

**Table:**

Refinement on *F*^2^	Secondary atom site location: difference Fourier map
Least-squares matrix: full	Hydrogen site location: mixed
*R*[*F*^2^ > 2σ(*F*^2^)] = 0.042	H-atom parameters constrained
*wR*(*F*^2^) = 0.108	*w* = 1/[σ^2^(*F*_o_^2^) + (0.0487*P*)^2^ + 1.5162*P*] where *P* = (*F*_o_^2^ + 2*F*_c_^2^)/3
*S* = 1.02	(Δ/σ)_max_ < 0.001
3240 reflections	Δρ_max_ = 0.32 e Å^−^^3^
184 parameters	Δρ_min_ = −0.19 e Å^−^^3^
0 restraints	Extinction correction: SHELXL-2019/2 (Sheldrick, 2008), Fc^*^=kFc[1+0.001xFc^2^λ^3^/sin(2θ)]^-1/4^
Primary atom site location: dual	Extinction coefficient: 0.0014 (4)

### (3aRS,4RS,9aSR)-3-Oxo-2-(propan-2-yl)-2,3,3a,4,9,9a-hexahydro-1H-benzo[f]isoindole-4-carboxylic acid (II) Special details

**Table:**

Geometry. All esds (except the esd in the dihedral angle between two l.s. planes) are estimated using the full covariance matrix. The cell esds are taken into account individually in the estimation of esds in distances, angles and torsion angles; correlations between esds in cell parameters are only used when they are defined by crystal symmetry. An approximate (isotropic) treatment of cell esds is used for estimating esds involving l.s. planes.

### (3aRS,4RS,9aSR)-3-Oxo-2-(propan-2-yl)-2,3,3a,4,9,9a-hexahydro-1H-benzo[f]isoindole-4-carboxylic acid (II) Fractional atomic coordinates and isotropic or equivalent isotropic displacement parameters (Å^2^)

**Table:**

	*x*	*y*	*z*	*U*_iso_*/*U*_eq_	
C1	0.60850 (7)	0.61571 (19)	0.29553 (10)	0.0232 (3)	
H1A	0.583765	0.518919	0.295989	0.028*	
H1B	0.592352	0.721854	0.305077	0.028*	
C2	0.68282 (7)	0.59480 (17)	0.36994 (9)	0.0192 (3)	
H2	0.704221	0.708128	0.382438	0.023*	
C3	0.70846 (7)	0.51448 (18)	0.46394 (10)	0.0224 (3)	
H3A	0.682432	0.411449	0.453086	0.027*	
H3B	0.703078	0.594238	0.502227	0.027*	
C4	0.78093 (7)	0.46863 (17)	0.51731 (10)	0.0215 (3)	
C5	0.81858 (8)	0.44082 (18)	0.61502 (10)	0.0258 (3)	
H5	0.798500	0.456195	0.646134	0.031*	
C6	0.88425 (8)	0.39156 (19)	0.66755 (11)	0.0291 (4)	
H6	0.908868	0.374215	0.733961	0.035*	
C7	0.91399 (8)	0.36760 (19)	0.62300 (11)	0.0303 (4)	
H7	0.958918	0.332208	0.658469	0.036*	
C8	0.87778 (7)	0.39557 (19)	0.52631 (11)	0.0267 (3)	
H8	0.898407	0.379403	0.496007	0.032*	
C9	0.81166 (7)	0.44700 (17)	0.47256 (10)	0.0208 (3)	
C10	0.77554 (7)	0.47589 (18)	0.36596 (10)	0.0199 (3)	
H10	0.783543	0.374453	0.340160	0.024*	
C11	0.70147 (7)	0.48662 (17)	0.31745 (9)	0.0184 (3)	
H11	0.686573	0.369028	0.316722	0.022*	
C12	0.65519 (7)	0.55150 (17)	0.21746 (10)	0.0198 (3)	
C13	0.80492 (7)	0.62868 (18)	0.35041 (10)	0.0227 (3)	
C14	0.54115 (7)	0.6643 (2)	0.11489 (10)	0.0276 (3)	
H10A	0.548602	0.645306	0.065779	0.033*	
C15	0.48583 (9)	0.5474 (3)	0.09262 (13)	0.0466 (5)	
H15A	0.499459	0.429709	0.096702	0.070*	
H15B	0.445963	0.570504	0.028408	0.070*	
H15C	0.476272	0.566514	0.138330	0.070*	
C16	0.52363 (9)	0.8486 (2)	0.11078 (13)	0.0441 (5)	
H16A	0.561092	0.919211	0.127077	0.066*	
H16B	0.513755	0.869581	0.156005	0.066*	
H16C	0.484672	0.876346	0.046602	0.066*	
N1	0.60273 (6)	0.62084 (15)	0.20737 (8)	0.0225 (3)	
O1	0.66190 (5)	0.53845 (13)	0.15330 (7)	0.0242 (3)	
O2	0.78009 (5)	0.77364 (13)	0.35226 (8)	0.0288 (3)	
H2O	0.802114	0.854226	0.350604	0.043*	
O3	0.84684 (7)	0.61777 (16)	0.33814 (11)	0.0513 (4)	

### (3aRS,4RS,9aSR)-3-Oxo-2-(propan-2-yl)-2,3,3a,4,9,9a-hexahydro-1H-benzo[f]isoindole-4-carboxylic acid (II) Atomic displacement parameters (Å^2^)

**Table:**

	*U*^11^	*U*^22^	*U*^33^	*U*^12^	*U*^13^	*U*^23^
C1	0.0273 (8)	0.0241 (8)	0.0252 (7)	0.0013 (6)	0.0195 (7)	−0.0003 (6)
C2	0.0241 (7)	0.0168 (7)	0.0218 (7)	−0.0006 (6)	0.0165 (6)	−0.0019 (6)
C3	0.0289 (8)	0.0214 (7)	0.0224 (7)	−0.0019 (6)	0.0183 (7)	−0.0017 (6)
C4	0.0299 (8)	0.0133 (7)	0.0224 (7)	−0.0037 (6)	0.0163 (6)	−0.0018 (6)
C5	0.0375 (9)	0.0177 (7)	0.0235 (7)	−0.0047 (6)	0.0191 (7)	−0.0026 (6)
C6	0.0360 (9)	0.0207 (8)	0.0194 (7)	−0.0068 (7)	0.0106 (7)	−0.0003 (6)
C7	0.0238 (8)	0.0277 (8)	0.0281 (8)	−0.0042 (6)	0.0094 (7)	0.0031 (7)
C8	0.0262 (8)	0.0246 (8)	0.0281 (8)	−0.0030 (6)	0.0156 (7)	0.0015 (6)
C9	0.0258 (8)	0.0146 (7)	0.0216 (7)	−0.0032 (6)	0.0140 (6)	−0.0006 (6)
C10	0.0240 (7)	0.0181 (7)	0.0208 (7)	0.0017 (6)	0.0150 (6)	0.0004 (6)
C11	0.0227 (7)	0.0156 (6)	0.0203 (7)	−0.0005 (5)	0.0147 (6)	−0.0006 (5)
C12	0.0237 (7)	0.0149 (7)	0.0224 (7)	0.0003 (5)	0.0148 (6)	−0.0013 (6)
C13	0.0243 (7)	0.0265 (8)	0.0216 (7)	0.0017 (6)	0.0161 (6)	0.0025 (6)
C14	0.0235 (8)	0.0359 (9)	0.0222 (7)	0.0048 (6)	0.0130 (7)	−0.0013 (6)
C15	0.0351 (10)	0.0659 (13)	0.0332 (9)	−0.0163 (9)	0.0173 (8)	−0.0145 (9)
C16	0.0356 (10)	0.0421 (11)	0.0364 (10)	0.0152 (8)	0.0119 (8)	0.0049 (8)
N1	0.0240 (7)	0.0254 (6)	0.0212 (6)	0.0041 (5)	0.0153 (5)	0.0014 (5)
O1	0.0306 (6)	0.0255 (6)	0.0223 (5)	0.0062 (4)	0.0190 (5)	0.0016 (4)
O2	0.0388 (6)	0.0192 (5)	0.0459 (7)	−0.0038 (4)	0.0348 (6)	−0.0010 (5)
O3	0.0619 (9)	0.0373 (7)	0.0955 (11)	0.0094 (6)	0.0690 (9)	0.0134 (7)

### (3aRS,4RS,9aSR)-3-Oxo-2-(propan-2-yl)-2,3,3a,4,9,9a-hexahydro-1H-benzo[f]isoindole-4-carboxylic acid (II) Geometric parameters (Å, º)

**Table:**

C1—N1	1.4705 (17)	C10—C11	1.5191 (19)
C1—C2	1.520 (2)	C10—C13	1.525 (2)
C1—H1A	0.9900	C10—H10	1.0000
C1—H1B	0.9900	C11—C12	1.5050 (19)
C2—C3	1.5101 (19)	C11—H11	1.0000
C2—C11	1.5252 (18)	C12—O1	1.2426 (16)
C2—H2	1.0000	C12—N1	1.3402 (17)
C3—C4	1.514 (2)	C13—O3	1.2005 (17)
C3—H3A	0.9900	C13—O2	1.3154 (17)
C3—H3B	0.9900	C14—N1	1.4666 (19)
C4—C5	1.398 (2)	C14—C16	1.512 (2)
C4—C9	1.4067 (19)	C14—C15	1.515 (2)
C5—C6	1.382 (2)	C14—H10A	1.0000
C5—H5	0.9500	C15—H15A	0.9800
C6—C7	1.384 (2)	C15—H15B	0.9800
C6—H6	0.9500	C15—H15C	0.9800
C7—C8	1.386 (2)	C16—H16A	0.9800
C7—H7	0.9500	C16—H16B	0.9800
C8—C9	1.395 (2)	C16—H16C	0.9800
C8—H8	0.9500	O2—H2O	0.8567
C9—C10	1.5358 (19)		
			
N1—C1—C2	101.87 (11)	C13—C10—C9	109.95 (11)
N1—C1—H1A	111.4	C11—C10—H10	107.6
C2—C1—H1A	111.4	C13—C10—H10	107.6
N1—C1—H1B	111.4	C9—C10—H10	107.6
C2—C1—H1B	111.4	C12—C11—C10	120.47 (11)
H1A—C1—H1B	109.3	C12—C11—C2	102.35 (11)
C3—C2—C1	119.24 (11)	C10—C11—C2	114.23 (11)
C3—C2—C11	110.09 (11)	C12—C11—H11	106.3
C1—C2—C11	101.86 (11)	C10—C11—H11	106.3
C3—C2—H2	108.4	C2—C11—H11	106.3
C1—C2—H2	108.4	O1—C12—N1	124.96 (13)
C11—C2—H2	108.4	O1—C12—C11	127.15 (12)
C2—C3—C4	110.29 (11)	N1—C12—C11	107.83 (11)
C2—C3—H3A	109.6	O3—C13—O2	123.31 (14)
C4—C3—H3A	109.6	O3—C13—C10	123.27 (13)
C2—C3—H3B	109.6	O2—C13—C10	113.41 (11)
C4—C3—H3B	109.6	N1—C14—C16	111.31 (13)
H3A—C3—H3B	108.1	N1—C14—C15	109.98 (14)
C5—C4—C9	118.36 (14)	C16—C14—C15	112.27 (15)
C5—C4—C3	118.63 (12)	N1—C14—H10A	107.7
C9—C4—C3	122.98 (12)	C16—C14—H10A	107.7
C6—C5—C4	121.76 (14)	C15—C14—H10A	107.7
C6—C5—H5	119.1	C14—C15—H15A	109.5
C4—C5—H5	119.1	C14—C15—H15B	109.5
C5—C6—C7	119.71 (14)	H15A—C15—H15B	109.5
C5—C6—H6	120.1	C14—C15—H15C	109.5
C7—C6—H6	120.1	H15A—C15—H15C	109.5
C6—C7—C8	119.56 (15)	H15B—C15—H15C	109.5
C6—C7—H7	120.2	C14—C16—H16A	109.5
C8—C7—H7	120.2	C14—C16—H16B	109.5
C7—C8—C9	121.35 (14)	H16A—C16—H16B	109.5
C7—C8—H8	119.3	C14—C16—H16C	109.5
C9—C8—H8	119.3	H16A—C16—H16C	109.5
C8—C9—C4	119.25 (13)	H16B—C16—H16C	109.5
C8—C9—C10	117.75 (12)	C12—N1—C14	123.11 (12)
C4—C9—C10	123.00 (13)	C12—N1—C1	112.46 (12)
C11—C10—C13	114.78 (11)	C14—N1—C1	123.31 (12)
C11—C10—C9	108.97 (11)	C13—O2—H2O	108.7
			
N1—C1—C2—C3	−155.24 (12)	C9—C10—C11—C2	44.17 (15)
N1—C1—C2—C11	−33.92 (13)	C3—C2—C11—C12	162.67 (11)
C1—C2—C3—C4	166.66 (12)	C1—C2—C11—C12	35.21 (13)
C11—C2—C3—C4	49.57 (15)	C3—C2—C11—C10	−65.43 (15)
C2—C3—C4—C5	161.10 (12)	C1—C2—C11—C10	167.11 (11)
C2—C3—C4—C9	−21.14 (18)	C10—C11—C12—O1	31.0 (2)
C9—C4—C5—C6	−0.7 (2)	C2—C11—C12—O1	159.03 (14)
C3—C4—C5—C6	177.20 (13)	C10—C11—C12—N1	−151.79 (12)
C4—C5—C6—C7	−0.4 (2)	C2—C11—C12—N1	−23.74 (14)
C5—C6—C7—C8	0.9 (2)	C11—C10—C13—O3	−140.85 (16)
C6—C7—C8—C9	−0.2 (2)	C9—C10—C13—O3	95.89 (18)
C7—C8—C9—C4	−0.9 (2)	C11—C10—C13—O2	39.50 (17)
C7—C8—C9—C10	179.68 (13)	C9—C10—C13—O2	−83.76 (14)
C5—C4—C9—C8	1.3 (2)	O1—C12—N1—C14	10.9 (2)
C3—C4—C9—C8	−176.47 (13)	C11—C12—N1—C14	−166.44 (12)
C5—C4—C9—C10	−179.29 (13)	O1—C12—N1—C1	179.09 (13)
C3—C4—C9—C10	3.0 (2)	C11—C12—N1—C1	1.79 (16)
C8—C9—C10—C11	165.75 (12)	C16—C14—N1—C12	−124.19 (15)
C4—C9—C10—C11	−13.68 (18)	C15—C14—N1—C12	110.74 (16)
C8—C9—C10—C13	−67.65 (16)	C16—C14—N1—C1	68.85 (18)
C4—C9—C10—C13	112.92 (14)	C15—C14—N1—C1	−56.23 (18)
C13—C10—C11—C12	42.86 (17)	C2—C1—N1—C12	20.97 (15)
C9—C10—C11—C12	166.65 (11)	C2—C1—N1—C14	−170.83 (12)
C13—C10—C11—C2	−79.61 (15)		

### (3aRS,4RS,9aSR)-3-Oxo-2-(propan-2-yl)-2,3,3a,4,9,9a-hexahydro-1H-benzo[f]isoindole-4-carboxylic acid (II) Hydrogen-bond geometry (Å, º)

*Cg*3 is the centroid of the C4–C9 rings.

**Table:**

*D*—H···*A*	*D*—H	H···*A*	*D*···*A*	*D*—H···*A*
O2—H2*O*···O1^i^	0.86	1.74	2.5885 (17)	171
C2—H2···O2	1.00	2.33	3.002 (2)	124
C3—H3*B*···O2^ii^	0.99	2.56	3.500 (2)	159
C16—H16*A*···O3^i^	0.98	2.55	3.531 (3)	176
C3—H3*A*···*Cg*3^iii^	0.99	2.68	3.6164 (16)	158

Symmetry codes: (i) −*x*+3/2, *y*+1/2, −*z*+1/2; (ii) −*x*+3/2, −*y*+3/2, −*z*+1; (iii) −*x*+3/2, −*y*+1/2, −*z*+1.

### (4RS)-3-Oxo-2-phenyl-2,3,4,9-tetrahydro-1H-benzo[f]isoindole-4-carboxylic acid (III) Crystal data

**Table:**

C_19_H_15_NO_3_	*F*(000) = 640
*M**_r_* = 305.32	*D*_x_ = 1.431 Mg m^−^^3^
Monoclinic, *P*2_1_/*c*	Mo *K*α radiation, λ = 0.71073 Å
*a* = 11.9915 (10) Å	Cell parameters from 1457 reflections
*b* = 7.1296 (6) Å	θ = 3.2–27.5°
*c* = 16.7967 (16) Å	µ = 0.10 mm^−^^1^
β = 99.338 (5)°	*T* = 100 K
*V* = 1417.0 (2) Å^3^	Needle, colourless
*Z* = 4	0.40 × 0.04 × 0.03 mm

### (4RS)-3-Oxo-2-phenyl-2,3,4,9-tetrahydro-1H-benzo[f]isoindole-4-carboxylic acid (III) Data collection

**Table:**

Bruker Kappa APEXII area-detector diffractometer	1938 reflections with *I* > 2σ(*I*)
φ and ω scans	*R*_int_ = 0.088
Absorption correction: multi-scan (SADABS; Krause *et al.*, 2015)	θ_max_ = 27.8°, θ_min_ = 3.1°
*T*_min_ = 0.842, *T*_max_ = 1.000	*h* = −15→15
13142 measured reflections	*k* = −9→8
3226 independent reflections	*l* = −21→21

### (4RS)-3-Oxo-2-phenyl-2,3,4,9-tetrahydro-1H-benzo[f]isoindole-4-carboxylic acid (III) Refinement

**Table:**

Refinement on *F*^2^	Primary atom site location: dual
Least-squares matrix: full	Secondary atom site location: difference Fourier map
*R*[*F*^2^ > 2σ(*F*^2^)] = 0.054	Hydrogen site location: inferred from neighbouring sites
*wR*(*F*^2^) = 0.128	H-atom parameters constrained
*S* = 1.00	*w* = 1/[σ^2^(*F*_o_^2^) + (0.0485*P*)^2^ + 0.4913*P*] where *P* = (*F*_o_^2^ + 2*F*_c_^2^)/3
3226 reflections	(Δ/σ)_max_ < 0.001
209 parameters	Δρ_max_ = 0.31 e Å^−^^3^
0 restraints	Δρ_min_ = −0.24 e Å^−^^3^

### (4RS)-3-Oxo-2-phenyl-2,3,4,9-tetrahydro-1H-benzo[f]isoindole-4-carboxylic acid (III) Special details

**Table:**

Geometry. All esds (except the esd in the dihedral angle between two l.s. planes) are estimated using the full covariance matrix. The cell esds are taken into account individually in the estimation of esds in distances, angles and torsion angles; correlations between esds in cell parameters are only used when they are defined by crystal symmetry. An approximate (isotropic) treatment of cell esds is used for estimating esds involving l.s. planes.

### (4RS)-3-Oxo-2-phenyl-2,3,4,9-tetrahydro-1H-benzo[f]isoindole-4-carboxylic acid (III) Fractional atomic coordinates and isotropic or equivalent isotropic displacement parameters (Å^2^)

**Table:**

	*x*	*y*	*z*	*U*_iso_*/*U*_eq_	
C1	0.67655 (19)	0.6704 (3)	0.52923 (14)	0.0135 (5)	
H1A	0.677388	0.550199	0.499836	0.016*	
H1B	0.715759	0.767277	0.501825	0.016*	
C2	0.55840 (19)	0.7292 (3)	0.53520 (14)	0.0121 (5)	
C3	0.46823 (19)	0.7638 (3)	0.46488 (13)	0.0121 (5)	
H3A	0.455998	0.648343	0.431850	0.015*	
H3B	0.493059	0.863982	0.430895	0.015*	
C4	0.35804 (19)	0.8212 (3)	0.49095 (14)	0.0120 (5)	
C5	0.2653 (2)	0.8560 (3)	0.43085 (15)	0.0145 (5)	
H5	0.273609	0.843410	0.375808	0.017*	
C6	0.1616 (2)	0.9085 (3)	0.44982 (15)	0.0169 (5)	
H6	0.098667	0.927258	0.408338	0.020*	
C7	0.1507 (2)	0.9336 (3)	0.53027 (15)	0.0200 (6)	
H7	0.080529	0.972954	0.543958	0.024*	
C8	0.2417 (2)	0.9012 (3)	0.59016 (15)	0.0192 (6)	
H8	0.233665	0.919964	0.644939	0.023*	
C9	0.34539 (19)	0.8413 (3)	0.57183 (14)	0.0133 (5)	
C10	0.44045 (18)	0.8008 (3)	0.64234 (14)	0.0136 (5)	
H10	0.455695	0.918657	0.674566	0.016*	
C11	0.54682 (19)	0.7469 (3)	0.61223 (14)	0.0123 (5)	
C12	0.65455 (19)	0.7023 (3)	0.66446 (14)	0.0131 (5)	
C13	0.40316 (19)	0.6495 (3)	0.69763 (14)	0.0154 (5)	
C14	0.83942 (19)	0.5785 (3)	0.63723 (14)	0.0137 (5)	
C15	0.92114 (19)	0.6081 (3)	0.58824 (15)	0.0155 (5)	
H15	0.903718	0.680544	0.540364	0.019*	
C16	1.0281 (2)	0.5312 (3)	0.60984 (16)	0.0193 (6)	
H16	1.083583	0.551149	0.576249	0.023*	
C17	1.0553 (2)	0.4258 (3)	0.67955 (16)	0.0219 (6)	
H17	1.129055	0.375083	0.694328	0.026*	
C18	0.9731 (2)	0.3957 (3)	0.72738 (15)	0.0200 (6)	
H18	0.990690	0.323139	0.775217	0.024*	
C19	0.8661 (2)	0.4695 (3)	0.70646 (15)	0.0178 (6)	
H19	0.810335	0.445974	0.739447	0.021*	
N1	0.72847 (16)	0.6501 (3)	0.61417 (11)	0.0140 (4)	
O1	0.67475 (14)	0.7100 (2)	0.73924 (10)	0.0175 (4)	
O2	0.39637 (15)	0.4801 (2)	0.66416 (10)	0.0210 (4)	
H2O	0.377299	0.401658	0.696782	0.031*	
O3	0.38111 (16)	0.6825 (2)	0.76358 (11)	0.0279 (5)	

### (4RS)-3-Oxo-2-phenyl-2,3,4,9-tetrahydro-1H-benzo[f]isoindole-4-carboxylic acid (III) Atomic displacement parameters (Å^2^)

**Table:**

	*U*^11^	*U*^22^	*U*^33^	*U*^12^	*U*^13^	*U*^23^
C1	0.0167 (12)	0.0128 (12)	0.0107 (12)	−0.0027 (9)	0.0017 (10)	0.0010 (9)
C2	0.0155 (12)	0.0065 (11)	0.0147 (13)	−0.0011 (9)	0.0036 (10)	0.0014 (9)
C3	0.0152 (12)	0.0122 (11)	0.0088 (12)	0.0007 (9)	0.0012 (9)	0.0012 (9)
C4	0.0136 (12)	0.0087 (11)	0.0135 (12)	−0.0026 (9)	0.0014 (9)	−0.0003 (9)
C5	0.0185 (13)	0.0126 (12)	0.0124 (12)	−0.0031 (10)	0.0032 (10)	0.0002 (10)
C6	0.0159 (13)	0.0169 (13)	0.0163 (14)	−0.0009 (10)	−0.0023 (10)	0.0024 (10)
C7	0.0131 (13)	0.0285 (15)	0.0196 (14)	0.0019 (10)	0.0056 (11)	0.0051 (11)
C8	0.0183 (13)	0.0257 (14)	0.0142 (13)	0.0010 (10)	0.0048 (11)	0.0022 (10)
C9	0.0147 (12)	0.0117 (11)	0.0139 (12)	−0.0006 (9)	0.0032 (10)	0.0008 (10)
C10	0.0138 (12)	0.0159 (12)	0.0111 (12)	0.0000 (9)	0.0024 (10)	0.0016 (9)
C11	0.0153 (12)	0.0114 (12)	0.0100 (12)	−0.0011 (9)	0.0012 (10)	0.0008 (9)
C12	0.0140 (12)	0.0116 (12)	0.0138 (13)	−0.0015 (9)	0.0030 (10)	0.0002 (9)
C13	0.0119 (12)	0.0215 (13)	0.0124 (13)	0.0026 (9)	0.0006 (10)	0.0015 (10)
C14	0.0127 (12)	0.0123 (12)	0.0151 (13)	0.0000 (9)	−0.0008 (10)	−0.0011 (10)
C15	0.0181 (13)	0.0159 (12)	0.0129 (13)	−0.0002 (10)	0.0035 (10)	0.0013 (10)
C16	0.0167 (13)	0.0187 (13)	0.0237 (14)	−0.0008 (10)	0.0068 (11)	−0.0024 (11)
C17	0.0163 (13)	0.0217 (14)	0.0260 (15)	0.0055 (10)	−0.0016 (11)	−0.0015 (11)
C18	0.0212 (14)	0.0221 (13)	0.0152 (13)	0.0044 (10)	−0.0011 (11)	0.0045 (11)
C19	0.0176 (13)	0.0209 (14)	0.0161 (13)	−0.0011 (10)	0.0057 (11)	0.0011 (10)
N1	0.0136 (10)	0.0173 (10)	0.0109 (10)	−0.0003 (8)	0.0016 (8)	0.0004 (8)
O1	0.0185 (9)	0.0216 (9)	0.0123 (9)	0.0023 (7)	0.0021 (7)	−0.0017 (7)
O2	0.0311 (11)	0.0161 (9)	0.0173 (10)	−0.0027 (8)	0.0084 (8)	0.0028 (7)
O3	0.0397 (12)	0.0307 (11)	0.0168 (10)	0.0002 (9)	0.0148 (9)	−0.0007 (8)

### (4RS)-3-Oxo-2-phenyl-2,3,4,9-tetrahydro-1H-benzo[f]isoindole-4-carboxylic acid (III) Geometric parameters (Å, º)

**Table:**

C1—N1	1.468 (3)	C10—C13	1.537 (3)
C1—C2	1.496 (3)	C10—H10	1.0000
C1—H1A	0.9900	C11—C12	1.474 (3)
C1—H1B	0.9900	C12—O1	1.241 (3)
C2—C11	1.329 (3)	C12—N1	1.371 (3)
C2—C3	1.487 (3)	C13—O3	1.203 (3)
C3—C4	1.514 (3)	C13—O2	1.330 (3)
C3—H3A	0.9900	C14—C19	1.392 (3)
C3—H3B	0.9900	C14—C15	1.394 (3)
C4—C5	1.398 (3)	C14—N1	1.419 (3)
C4—C9	1.398 (3)	C15—C16	1.388 (3)
C5—C6	1.384 (3)	C15—H15	0.9500
C5—H5	0.9500	C16—C17	1.385 (4)
C6—C7	1.390 (3)	C16—H16	0.9500
C6—H6	0.9500	C17—C18	1.386 (4)
C7—C8	1.378 (3)	C17—H17	0.9500
C7—H7	0.9500	C18—C19	1.379 (3)
C8—C9	1.396 (3)	C18—H18	0.9500
C8—H8	0.9500	C19—H19	0.9500
C9—C10	1.532 (3)	O2—H2O	0.8400
C10—C11	1.497 (3)		
			
N1—C1—C2	102.67 (18)	C9—C10—C13	110.27 (19)
N1—C1—H1A	111.2	C11—C10—H10	107.9
C2—C1—H1A	111.2	C9—C10—H10	107.9
N1—C1—H1B	111.2	C13—C10—H10	107.9
C2—C1—H1B	111.2	C2—C11—C12	109.8 (2)
H1A—C1—H1B	109.1	C2—C11—C10	125.6 (2)
C11—C2—C3	125.5 (2)	C12—C11—C10	124.5 (2)
C11—C2—C1	110.0 (2)	O1—C12—N1	126.6 (2)
C3—C2—C1	124.6 (2)	O1—C12—C11	127.0 (2)
C2—C3—C4	111.79 (19)	N1—C12—C11	106.48 (19)
C2—C3—H3A	109.3	O3—C13—O2	124.0 (2)
C4—C3—H3A	109.3	O3—C13—C10	123.3 (2)
C2—C3—H3B	109.3	O2—C13—C10	112.72 (19)
C4—C3—H3B	109.3	C19—C14—C15	119.3 (2)
H3A—C3—H3B	107.9	C19—C14—N1	120.5 (2)
C5—C4—C9	119.0 (2)	C15—C14—N1	120.1 (2)
C5—C4—C3	117.9 (2)	C16—C15—C14	119.6 (2)
C9—C4—C3	123.1 (2)	C16—C15—H15	120.2
C6—C5—C4	121.4 (2)	C14—C15—H15	120.2
C6—C5—H5	119.3	C17—C16—C15	121.1 (2)
C4—C5—H5	119.3	C17—C16—H16	119.5
C5—C6—C7	119.2 (2)	C15—C16—H16	119.5
C5—C6—H6	120.4	C16—C17—C18	118.8 (2)
C7—C6—H6	120.4	C16—C17—H17	120.6
C8—C7—C6	120.0 (2)	C18—C17—H17	120.6
C8—C7—H7	120.0	C19—C18—C17	120.9 (2)
C6—C7—H7	120.0	C19—C18—H18	119.6
C7—C8—C9	121.2 (2)	C17—C18—H18	119.6
C7—C8—H8	119.4	C18—C19—C14	120.3 (2)
C9—C8—H8	119.4	C18—C19—H19	119.9
C8—C9—C4	119.1 (2)	C14—C19—H19	119.9
C8—C9—C10	117.7 (2)	C12—N1—C14	126.9 (2)
C4—C9—C10	123.2 (2)	C12—N1—C1	110.92 (18)
C11—C10—C9	110.81 (19)	C14—N1—C1	122.12 (18)
C11—C10—C13	111.80 (18)	C13—O2—H2O	109.5
			
N1—C1—C2—C11	2.2 (2)	C13—C10—C11—C12	−57.7 (3)
N1—C1—C2—C3	−178.22 (19)	C2—C11—C12—O1	177.6 (2)
C11—C2—C3—C4	−0.6 (3)	C10—C11—C12—O1	−3.2 (4)
C1—C2—C3—C4	179.9 (2)	C2—C11—C12—N1	−2.4 (3)
C2—C3—C4—C5	−179.24 (19)	C10—C11—C12—N1	176.7 (2)
C2—C3—C4—C9	1.0 (3)	C11—C10—C13—O3	128.6 (2)
C9—C4—C5—C6	−0.4 (3)	C9—C10—C13—O3	−107.7 (3)
C3—C4—C5—C6	179.8 (2)	C11—C10—C13—O2	−52.4 (3)
C4—C5—C6—C7	2.3 (3)	C9—C10—C13—O2	71.4 (2)
C5—C6—C7—C8	−1.7 (4)	C19—C14—C15—C16	−1.0 (4)
C6—C7—C8—C9	−0.7 (4)	N1—C14—C15—C16	−177.5 (2)
C7—C8—C9—C4	2.6 (4)	C14—C15—C16—C17	−0.3 (4)
C7—C8—C9—C10	−177.5 (2)	C15—C16—C17—C18	0.9 (4)
C5—C4—C9—C8	−2.0 (3)	C16—C17—C18—C19	−0.3 (4)
C3—C4—C9—C8	177.8 (2)	C17—C18—C19—C14	−1.0 (4)
C5—C4—C9—C10	178.1 (2)	C15—C14—C19—C18	1.6 (4)
C3—C4—C9—C10	−2.2 (3)	N1—C14—C19—C18	178.1 (2)
C8—C9—C10—C11	−177.4 (2)	O1—C12—N1—C14	6.1 (4)
C4—C9—C10—C11	2.5 (3)	C11—C12—N1—C14	−173.8 (2)
C8—C9—C10—C13	58.3 (3)	O1—C12—N1—C1	−176.2 (2)
C4—C9—C10—C13	−121.8 (2)	C11—C12—N1—C1	3.9 (2)
C3—C2—C11—C12	−179.5 (2)	C19—C14—N1—C12	32.1 (3)
C1—C2—C11—C12	0.0 (3)	C15—C14—N1—C12	−151.4 (2)
C3—C2—C11—C10	1.4 (4)	C19—C14—N1—C1	−145.4 (2)
C1—C2—C11—C10	−179.1 (2)	C15—C14—N1—C1	31.0 (3)
C9—C10—C11—C2	−2.1 (3)	C2—C1—N1—C12	−3.8 (2)
C13—C10—C11—C2	121.3 (2)	C2—C1—N1—C14	174.04 (19)
C9—C10—C11—C12	178.9 (2)		

### (4RS)-3-Oxo-2-phenyl-2,3,4,9-tetrahydro-1H-benzo[f]isoindole-4-carboxylic acid (III) Hydrogen-bond geometry (Å, º)

*Cg*1, *Cg*2, *Cg*3 and *Cg*4 are the centroids of the N1/C1/C2/C11/C12, C2–C11 C4–C9 and C16–C21 rings, respectively.

**Table:**

*D*—H···*A*	*D*—H	H···*A*	*D*···*A*	*D*—H···*A*
O2—H2*O*···O1^i^	0.84	1.90	2.741 (2)	174
C1—H1*A*···*Cg*2^ii^	0.99	2.91	3.756 (2)	144
C1—H1*B*···*Cg*3^iii^	0.99	2.58	3.427 (2)	144
C3—H3*A*···*Cg*1^ii^	0.99	2.70	3.593 (2)	150
C3—H3*B*···*Cg*2^iii^	0.99	2.60	3.388 (2)	137
C18—H18···*Cg*4^iv^	0.95	2.61	3.452 (2)	148

Symmetry codes: (i) −*x*+1, *y*−1/2, −*z*+3/2; (ii) −*x*+1, −*y*+1, −*z*+1; (iii) −*x*+1, −*y*+2, −*z*+1; (iv) −*x*+2, *y*−1/2, −*z*+3/2.
