# Supplementary material for: Consistent supra­molecular motif of C(7) O—H⋯O hydrogen-bonded chains and different local symmetries in three iso­indole-4-carb­oxy­lic acid derivatives
Source: Acta Crystallogr E Crystallogr Commun. 2026 Jan 1;82(Pt 1):40–6. doi: 10.1107/S2056989025010709 (PMC12810269; doi:10.1107/S2056989025010709)
Supplement: Supplementary file 8 [file e-82-00040-sup8.pdf]

Supplementary Material

Syntheses, crystal structures and Hirshfeld surface analyses of (3a*RS*,4*RS*, 9a*SR*)-3-oxo-2-(2-phenylethyl)-2,3,3a,4,9,9a-hexahydro- 1*H*-benzo[*f*]isoindole-4-carboxylic acid, (3a*RS*,4*RS*, 9a*SR*)-3-oxo-2-(propan-2-yl)-2,3,3a,4,9,9a-hexahydro- 1*H*-benzo[*f*]isoindole-4-carboxylic acid and (4*RS*)-3-oxo-2-phenyl-2,3,4,9-tetrahydro- 1*H*-benzo[*f*]isoindole-4-carboxylic acid

Kseniia A. Alekseeva,[a] Atash V. Gurbanov,[b] Ekaterina N. Tsulina,[a] Alexandra S. Golubenkova,[a] Mehmet Akkurt[c] and Gizachew Mulugeta Manahelohe[d]*

[a]RUDN University, 6 Miklukho-Maklaya St., Moscow 117198, Russian Federation, [b]Excellence Center, Baku State University, Z. Khalilov Str., AZ 33, Baku, Azerbaijan, [c]Department of Physics, Faculty of Sciences, Erciyes University, 38039 Kayseri, Turkey, and [d]Department of Chemistry, University of Gondar, P.O. Box 196, Gondar, Ethiopia

Correspondence e-mail: **Gizachew.Mulugeta@uog.edu.et**

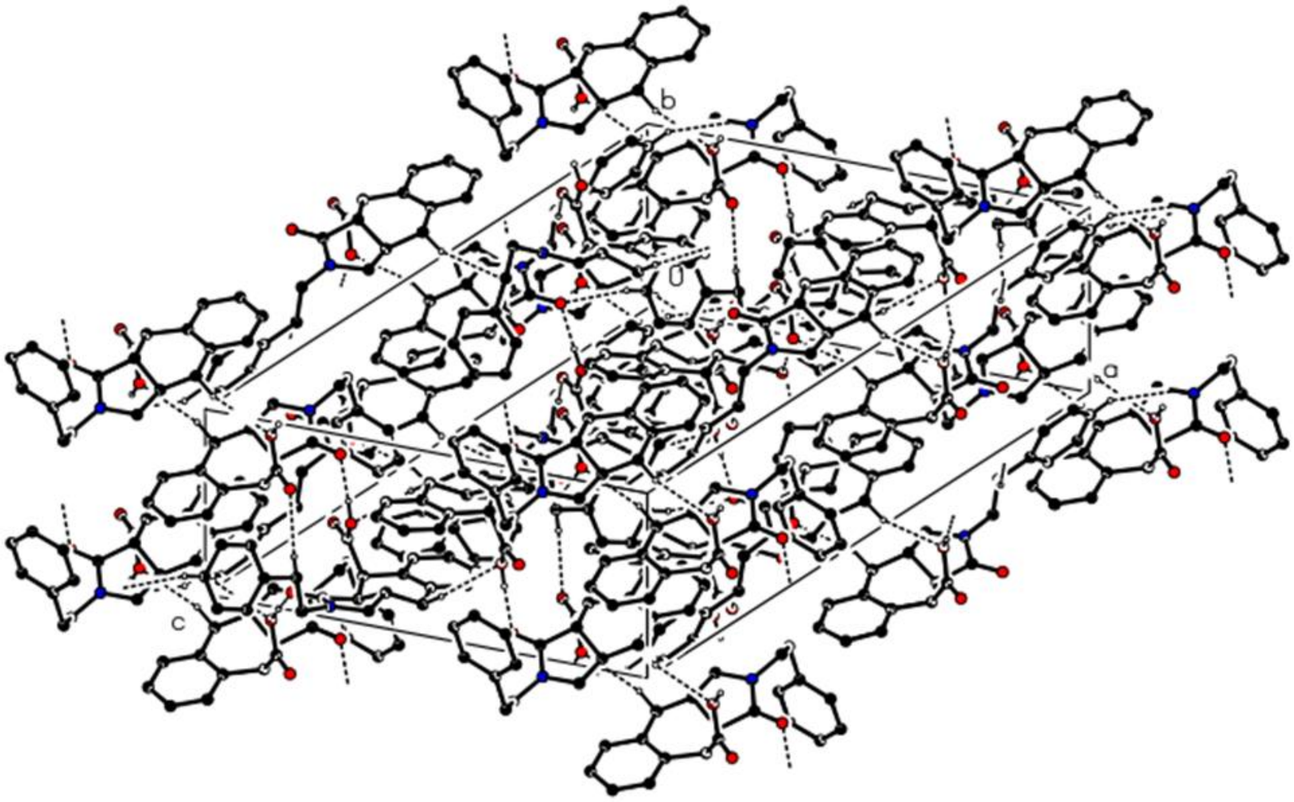

Figure S1. A general view of the C---H...O and O---H...O interactions of (**I**) in the unitcell. H atoms with non-hydrogen bonding were omitted for clarity.

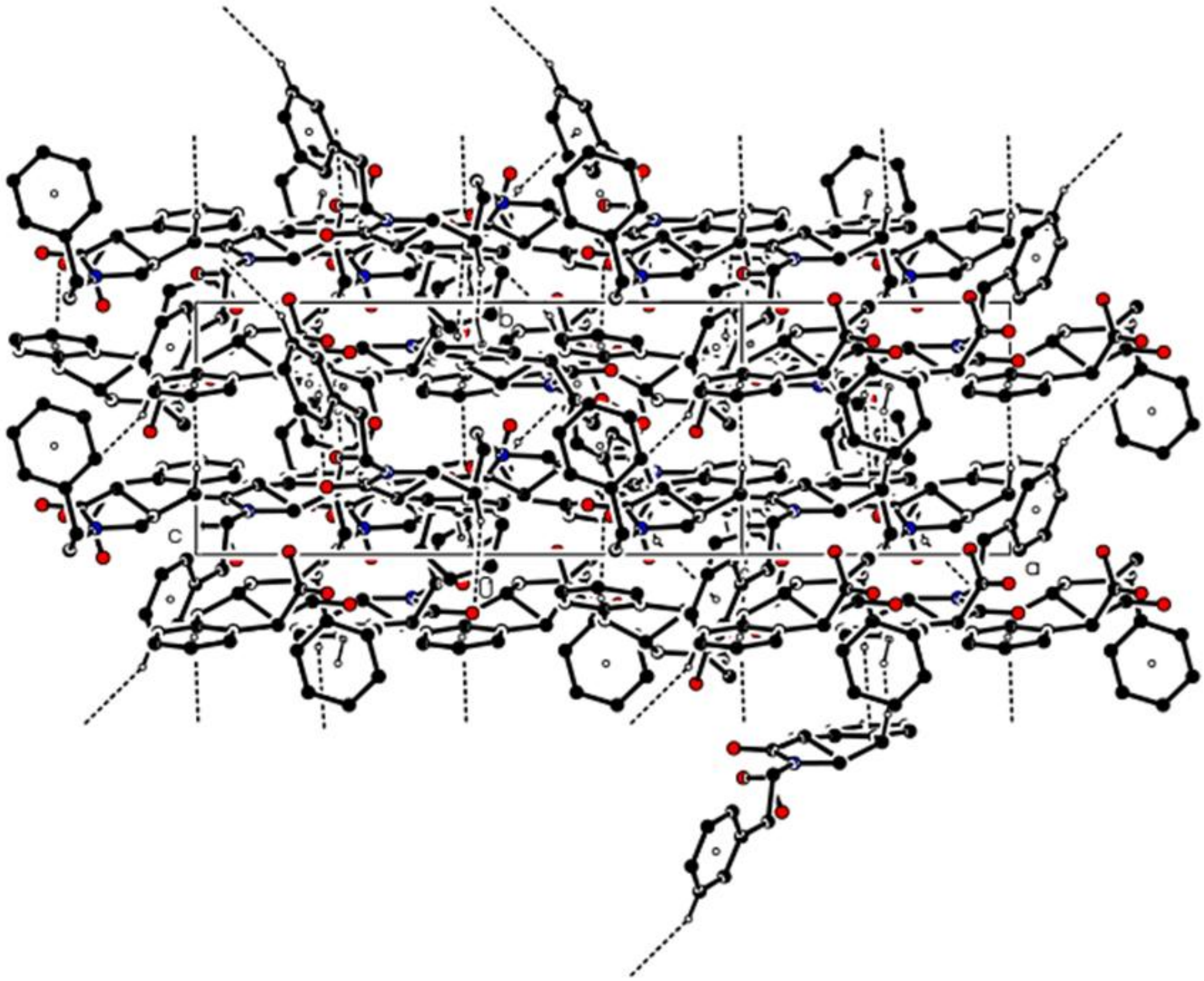

Figure S2. View of the C---H...π and C---O... π interactions of (**I**) along the c-axis. H atoms with non- hydrogen bonding were omitted for clarity.

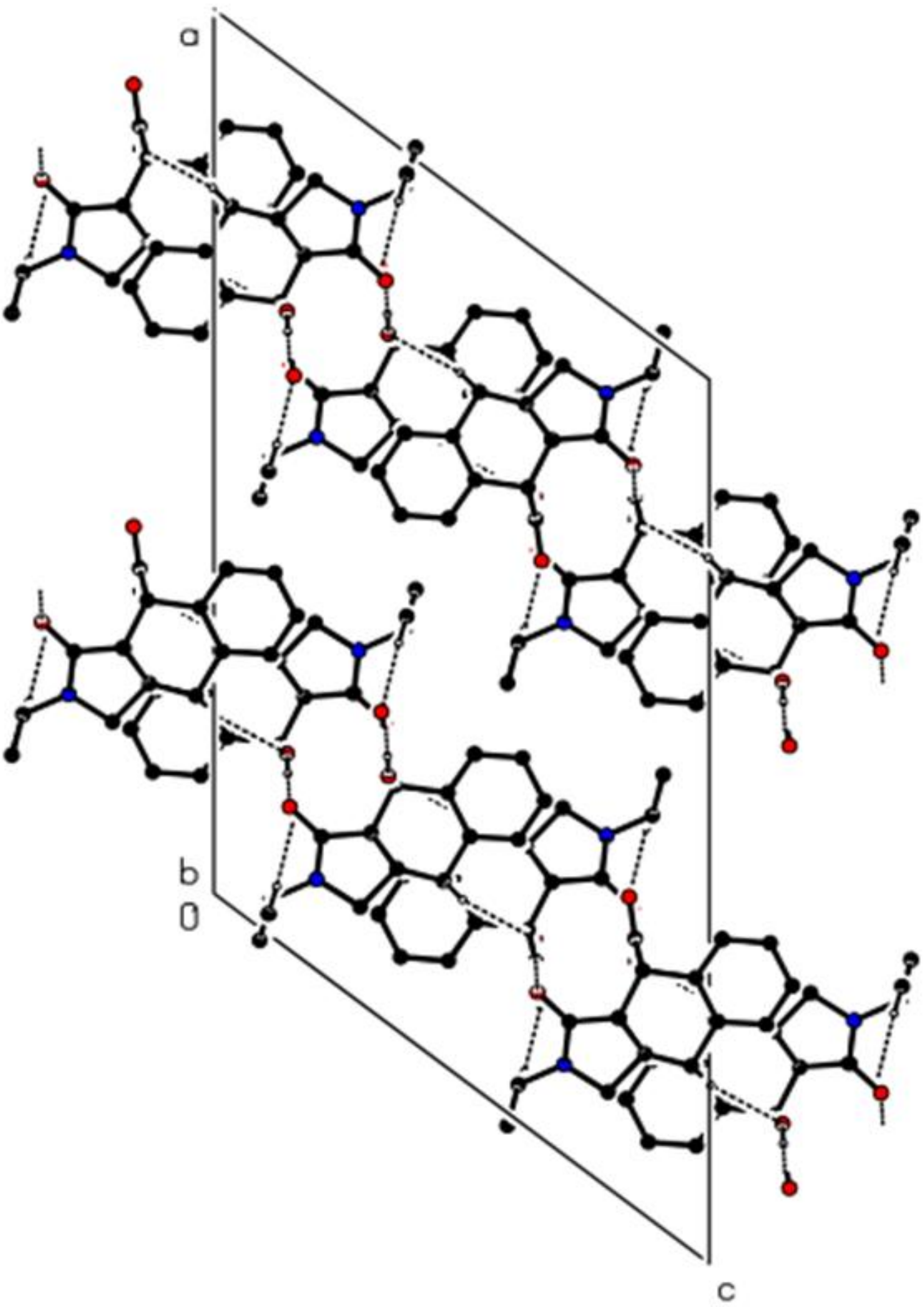

Figure S3. View of the C---H...O and O...H...O interactions of (**II**) along the b-axis. H atoms with non- hydrogen bonding were omitted for clarity.

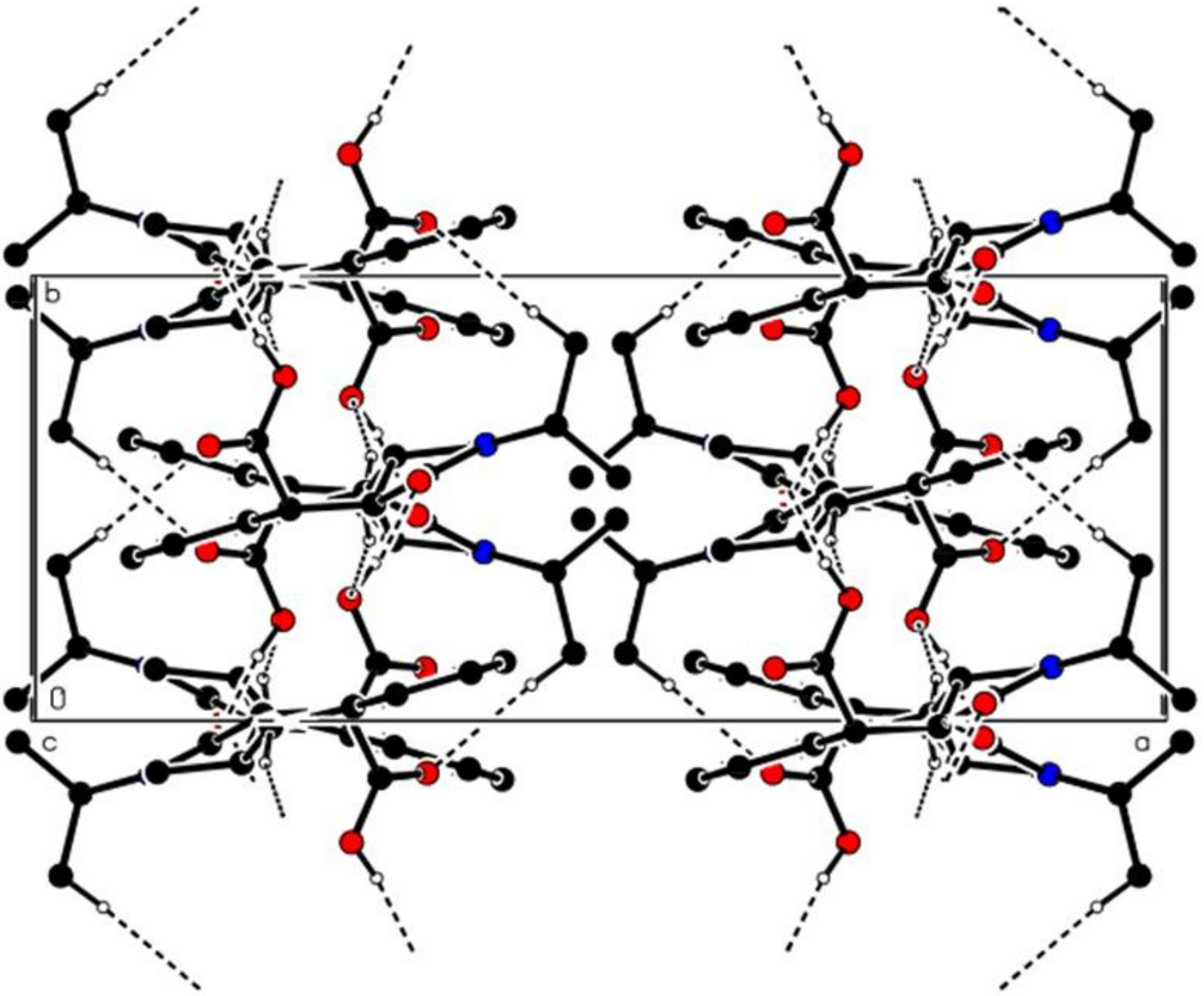

Figure S4. View of the C---H...O and O...H...O interactions of (**II**) along the c-axis. H atoms with non- hydrogen bonding were omitted for clarity.

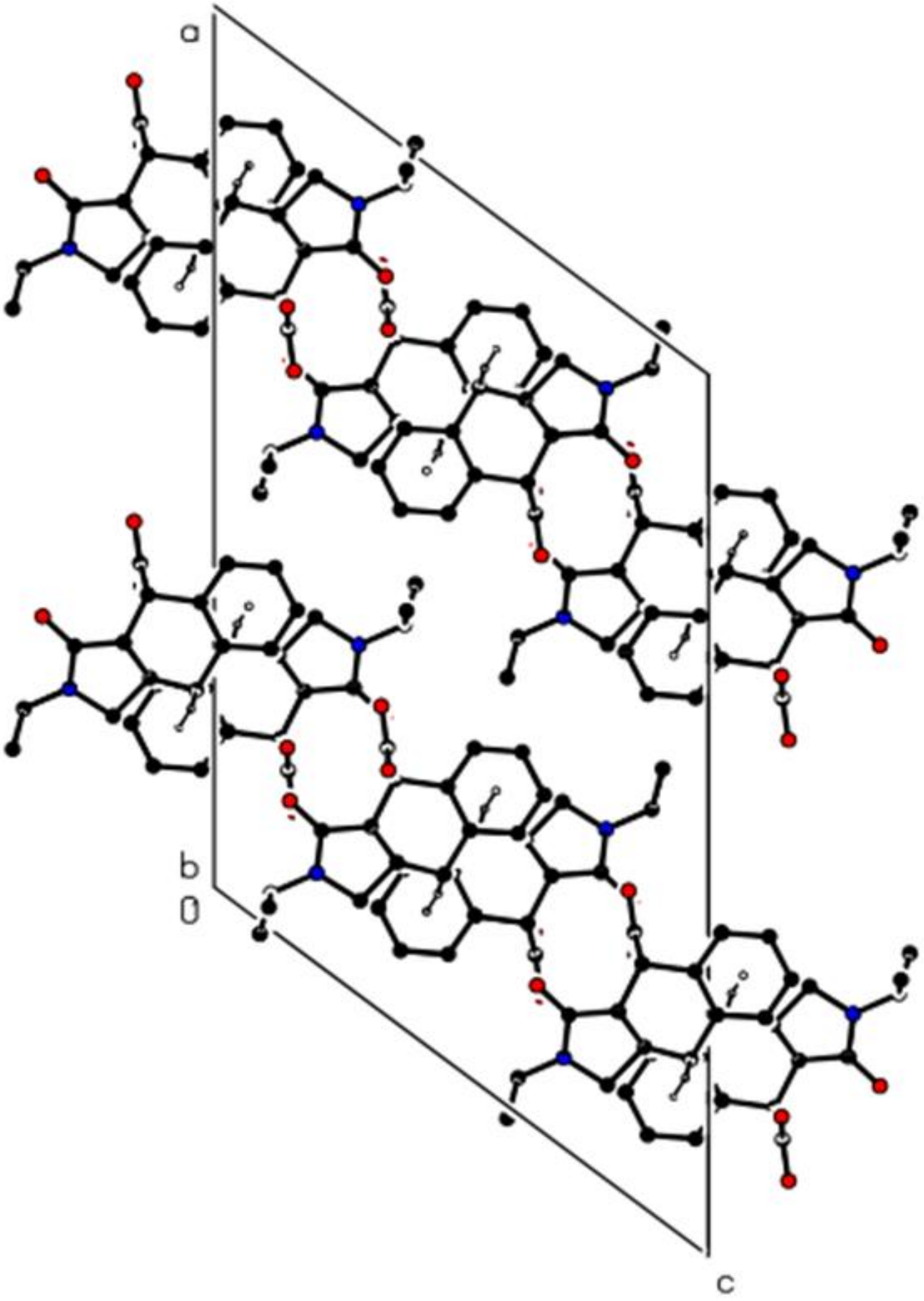

Figure S5. View of the C—H···π interactions of (**II**) along the b-axis. H atoms with non- hydrogen bonding were omitted for clarity.

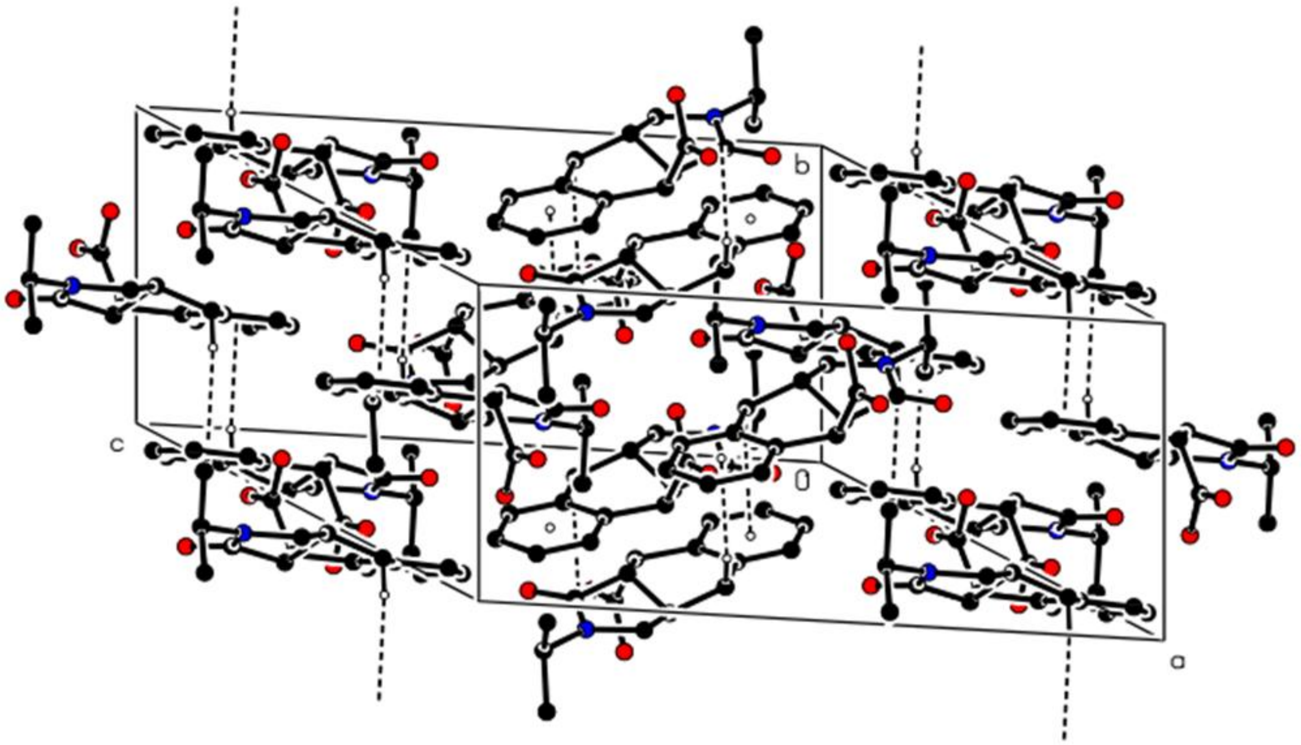

Figure S6. A general view of the C—H···π interactions of (**II**) in the unitcell. H atoms with non- hydrogen bonding were omitted for clarity.

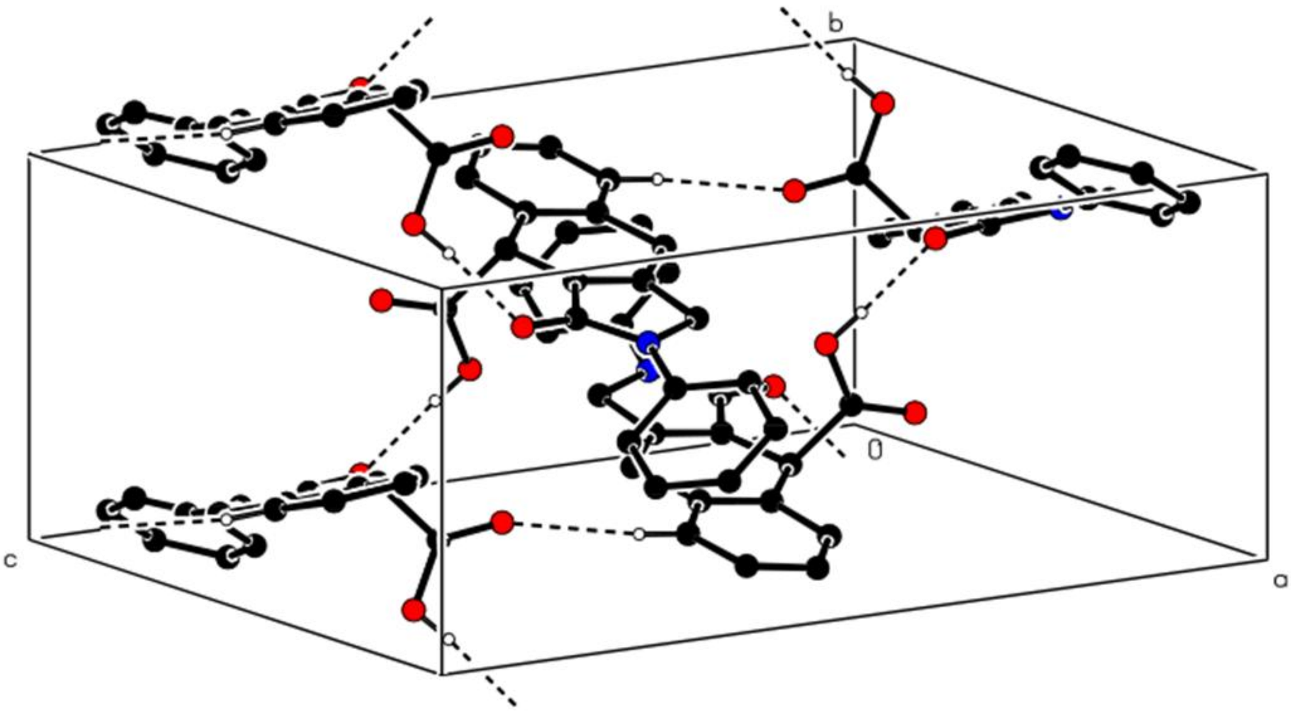

Figure S7. A general view of the O---H...O and C---H...O interactions of (**III**) in the unit cell. H atoms with non-hydrogen bonding were omitted for clarity.

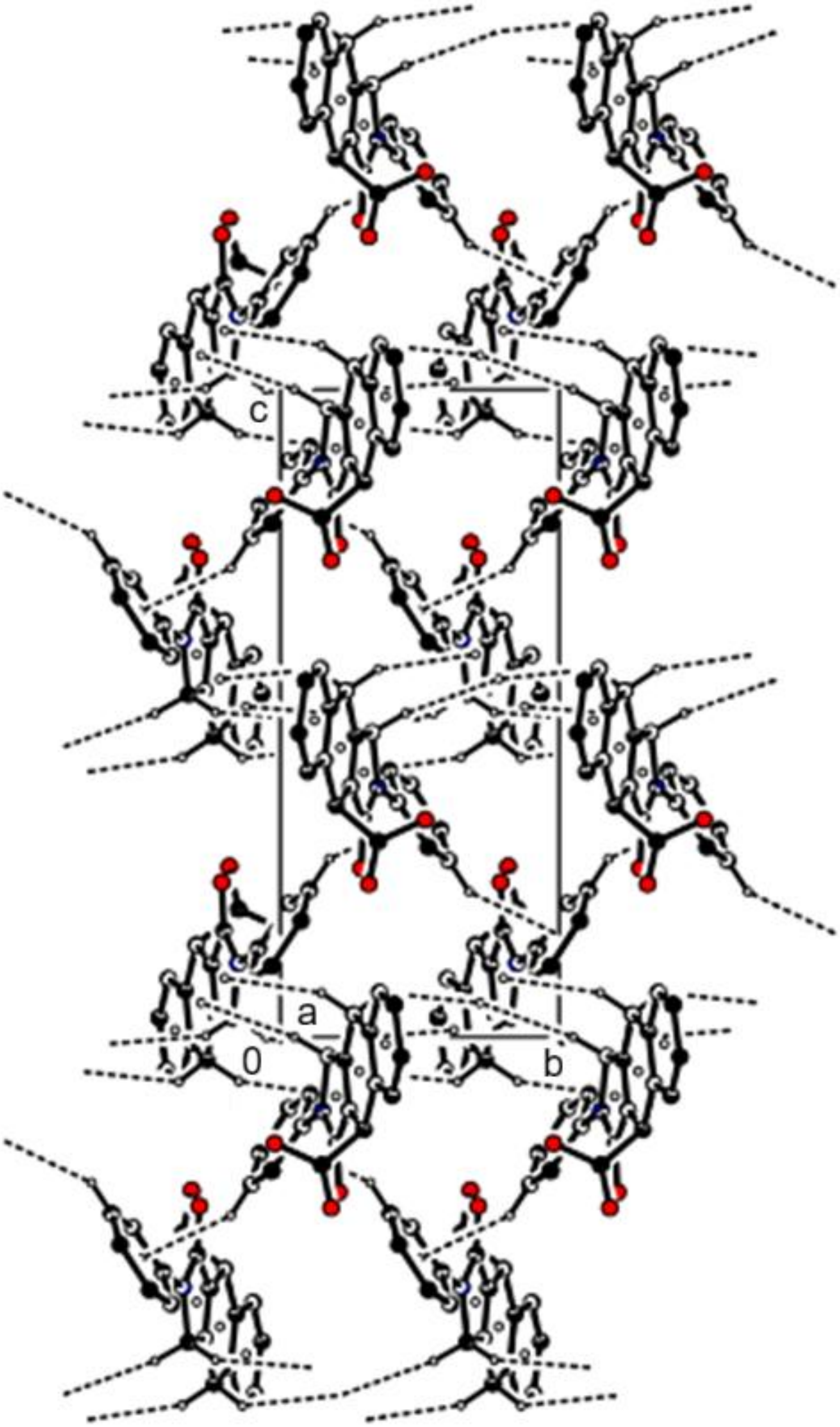

Figure S8. View of the C—H⋯π interactions of (**III**) along the a-axis. H atoms with non- hydrogen bonding were omitted for clarity.

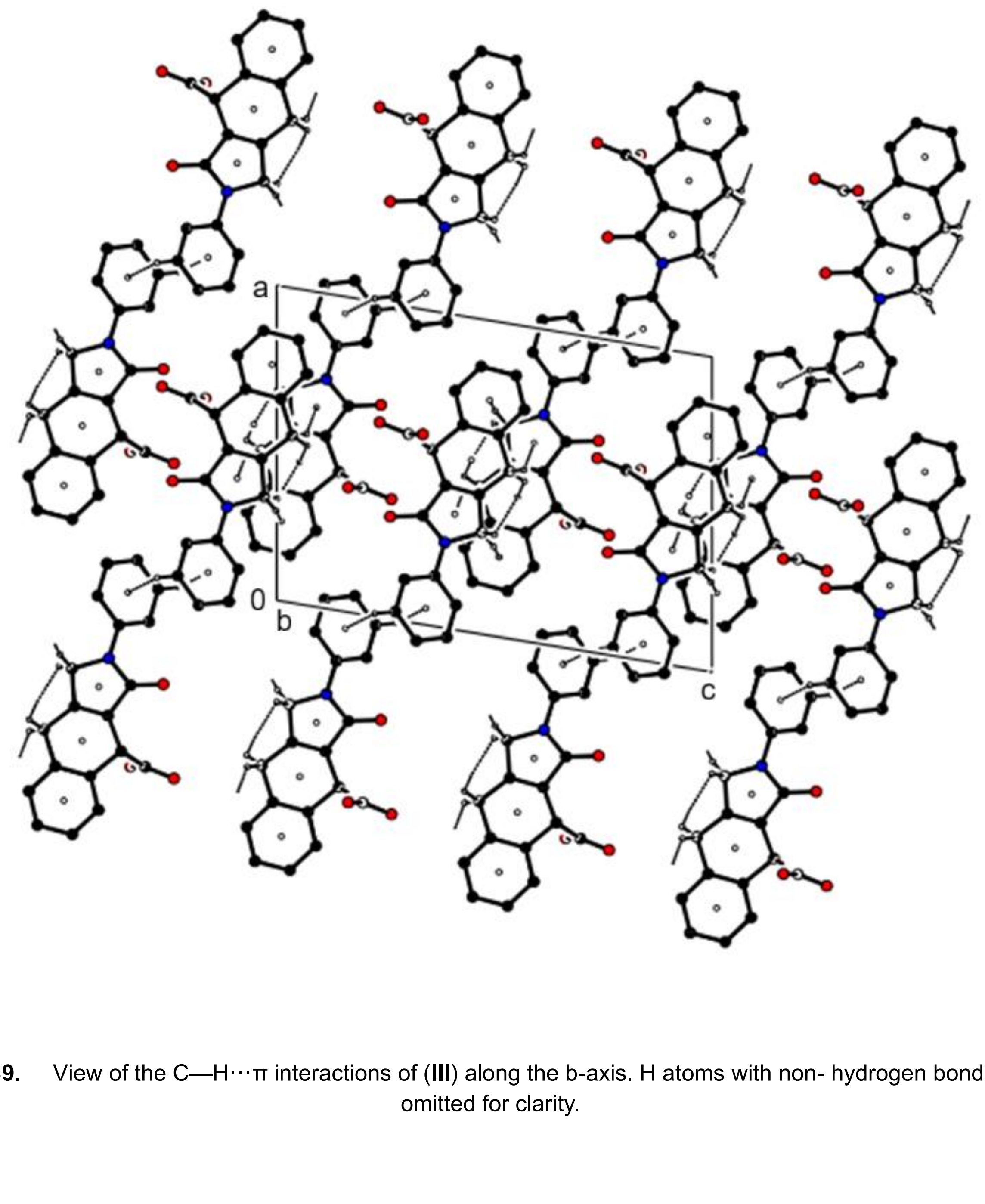

Figure S9. View of the C—H···π interactions of (**III**) along the b-axis. H atoms with non- hydrogen bonding were omitted for clarity.
